# Principal component and discriminant analyses as powerful tools to support taxonomic identification and their use for functional and phylogenetic signal detection of isolated fossil shark teeth

**DOI:** 10.1371/journal.pone.0188806

**Published:** 2017-11-28

**Authors:** Giuseppe Marramà, Jürgen Kriwet

**Affiliations:** Department of Palaeontology, University of Vienna, Vienna, Austria; Universita degli Studi di Urbino Carlo Bo, ITALY

## Abstract

Identifying isolated teeth of fossil selachians only based on qualitative characters is sometimes hindered by similarity in their morphology, resulting often in heated taxonomic debates. On the other hand, the use of quantitative characters (i.e. measurements) has been often neglected or underestimated in characterization and identification of fossil teeth of selachians. Here we show that, employing a robust methodological protocol based on principal component and discriminant analyses on a sample of 175 isolated fossil teeth of lamniform sharks, the traditional morphometrics can be useful to support and complement the classic taxonomic identification made on qualitative features. Furthermore, we show that discriminant analysis can be successfully useful to assign indeterminate isolated shark teeth to a certain taxon. Finally, the degree of separation of the clusters might be used to predict functional and probably also phylogenetic signals in lamniform shark teeth. However, this needs to be tested in the future employing teeth of more extant and extinct lamniform sharks and it must be pointed out that this approach does not replace in any way the qualitative analysis, but it is intended to complement and support it.

## Introduction

The fossil record of elasmobranch fishes (sharks, rays, skates) is mainly represented by isolated teeth, which occur in marine, brackish and freshwater sediments worldwide ranging from Devonian to Recent [1]. Although only a few exceptional fossiliferous deposits yielded complete articulated chondrichthyan fishes, e.g. Devonian Cleveland Shale in Ohio, Early Jurassic of Lyme Regis in England, Late Jurassic of southern Germany, Late Cretaceous of Lebanon, Paleogene localities in Italy (Bolca), Germany (Grube Unterfeld Lagerstätte), and North America (Green River Formation) [2–6], which are of outmost importance to understand their evolutionary trends, isolated teeth are often the only morphological remains that can be used for taxonomic and systematic purposes and to interpret evolutionary trajectories in fossil elasmobranchs [1]. The number of publications on fossil sharks steadily increased since the seminal work “Researches sur les poissons fossiles” published by Louis Agassiz [7, 8]. However, with the increasing number of taxa identified and erected based on teeth alone so far, the problem of the taxonomic identification based on qualitative dental characters alone is ever more increasing, mainly because several lineages often show similar morphological traits that are difficult to quantify resulting in unidentified convergent patterns (compare e.g. Cappetta’s treatises [1, 9]). This resulted several times in quite controversial discussions about the taxonomic identity of fossil taxa. For example, although there is remarkable disparity in the morphology of fossil teeth in lamniform sharks, most of the fossil taxa traditionally included within the family Odontaspididae are difficult to classify at genus level. Recently, e.g., Purdy & Francis [10] questioned the validity of the extinct sand tiger shark *Brachycarcharias* Cappetta & Nolf, 2005, a shark widely spread across the northern and southern hemispheres during the early Paleogene [11], stating that there are no robust morphological evidences to create a new genus for the species *Lamna lerichei* Casier, 1946. However, as pointed out by Cappetta [1] the authors never tested the validity of this taxon in comparison with previously described taxa, which are considered synonymous. Another study, although assuming *Brachycarcharias* to be valid, hypothesized that *B*. *lerichei* may have had similar feeding and habitat preferences as the living porbeagle shark *Lamna nasus* because of their similar tooth morphology [12].

Since most of the fossil lamniforms have no modern equivalents, the vast majority of taxa is much more difficult to assign to a specific genus and often do not fit the dental design of living genera, rendering any interpretation about their phylogenetic relationships difficult [1, 13]. In this perspective, we expect that quantitative analyses of tooth features can help to support qualitative identifications, solving most of the taxonomic issues, and to hypothesize relationships among taxa, since the dental morphology of a taxon is a complex result of different processes, including evolutionary processes [1].

The use of biometric characters (i.e. measurements) for tooth characterization has been often neglected or underestimated in fossil selachian taxonomy, and identification based on qualitative and sometimes few or a restricted number of morphological characters (overall shape, relative size, relative bending of the cusps) has been preferred so far. Only few studies including isolated fossil elasmobranch teeth attempted quantitative approaches for taxonomic purposes, mostly using geometric morphometrics [14–16]. More recently, Belben *et al*. [17] investigated the morphospace occupation of sharks based on tooth measurements, in order to investigate ecological replacement of top predators occurring in Moroccan marine settings after the end-Cretaceous extinction.

The use of multivariate statistical techniques as principal component (PCA) and discriminant (DA) analyses based on a large set of different quantifiable variables (pure linear measurements and angles), however, has never been used for taxonomic purposes and to identify phylogenetic signals of isolated fossil shark teeth using both living and fossil taxa for comparisons employing a robust protocol and statistics for testing the validity of the method. The goal of this paper is therefore to propose and encourage the use of traditional morphometric methodologies, testing the results with robust statistical analyses, in order to provide additional support for identifying fossil shark teeth based on qualitative features, to solve contradicting taxonomic issues, and to hypothesize phylogenetic relationships among fossil and living taxa. For this purpose we use isolated teeth of several fossil and living representatives of lamniform sharks. The method employed here, nevertheless, can be also adopted and modified to analyse the taxonomy and phylogenetic relationships in other lineages.

## Materials and methods

### Taxon sampling

In this study, the analyses were performed at genus level using one to four species as representatives of the morphology of each genus. We used this rank as standard unit for two main reasons: 1) the genus is considered as a reliable taxonomic rank in biological and palaeobiological analyses, since the concept of fossil species does not always correspond to the biological one [18, 19]. For this reason, the use of the genus as taxonomic unit has been widely preferred with respect to the species level in several papers focussing on biological analyses [20–23]. 2) The majority of the elasmobranch fossil species is difficult to identify below the generic level since most of the quantitative characters (e.g. sizes, proportions, shape, inclination of the cusp) useful to describe the different parts of a tooth (e.g. main cusp, lateral casplets, root, etc) are useful in taxonomy to identify mainly the genus. On the contrary, most of the features to discriminate fossil species are mainly based on qualitative features (e.g. presence of lingual folds, ornamentation, serrations), which cannot be used in a morphometric approach [1].

The present study is based on a sample of 175 isolated teeth, which previously have been used in several studies of fossil elasmobranchs [11, 24] or photographed in museum collections for the first time (S1 Table). This provides a well-established and reliable taxonomic frame for testing our results. The genus-level data set is based on a sample of five fossil species of three genera (*Carcharias acutissima*, *C*. *cuspidata*, *C*. *gustrowensis*, *Brachycarcharias lerichei*, and *Carcharomodus escheri*; 5, 13, 1, 40 and 16 specimens, respectively) and complete tooth series (including upper and lower teeth) of the extant portbeagle shark, *Lamna nasus*, and the sand tiger shark, *Carcharias taurus* (70 and 30 specimens, respectively). These two living taxa are used for comparisons and as control taxa, because the actual jaw position of each tooth is well known. For extant taxa, we excluded only teeth from the lateral-most positions (those beyond the seventh lateral tooth position) and intermediate teeth since they are not represented in the fossil sample that was examined. We selected lamniform genera since the similarities in their general tooth morphology can be used to test the power of our approach in detecting also minimal morphological differences and, at the same time, these taxa can be used as examples for solving taxonomic debates (see Introduction). Furthermore, three indeterminate fossil teeth of lamniform sharks from a single stratigraphic unit were included in our analyses to assess if multivariate methods are useful to properly identify teeth based on morphometry and assign them to a certain taxon.

For this study we only used measurements of the labial and lingual sides since these are often the only accessible sides in fossil specimens when the teeth still are embedded in and strictly associated to the sediment and bureaucratic rules of museum collections do not allow to extract them from the matrix (this is the case, for example, for specimens from the Bolca and Frauenweiler Lagerstätten, which also are included in this study). All fossil teeth were previously assigned to their respective positions using qualitative characters observed in *Brachycarcharias*, *Carcharias*, and *Carcharomodus* [1, 11, 24, 25], and subsequently compared with the results of the morphometric approach.

### Multivariate analyses

In this study we adopted two multivariate approaches. The principal component analysis (PCA) is one of the most often used multivariate statistical methods for investigating biological patterns and models based on large sets of correlated variables. PCA uses orthogonal transformation to convert multiple variables into a set of orthogonal uncorrelated axes, also called principal components (or PCs), which account for as much as possible of the variance in multivariate data sets, therefore reducing it to only few variables [26–33]. This approach has been widely used in various biological and ecological studies. In particular, this method was used to solve taxonomic issues in zoology [34–37], botany [38–41], microbiology [42], as well as for identifying niche dynamics through the analysis of morphological or ecological traits [33, 43, 44]. In palaeontology, PCA was successfully employed to support population or taxon separations [6, 45, 46], to avoid taxon over-splitting [47, 48], or to investigate biotic turnovers [17].

Discriminant analysis (DA) is used for testing hypotheses of morphologic similarities or differences employing pairwise comparisons between two groups, by projecting a multivariate data set down to one dimension and maximizing separation between groups separated a priori [32, 49]. Discriminant analysis is therefore useful for testing hypotheses of morphological similarities, and a significant 90% or greater separation between two groups is considered sufficient to support the presence of two different morphotypes [50]. In palaeontology, this multivariate approach previously has been used, for example, to test variations within a single population and to identify isolated dinosaur teeth [51]. Other than being a support for PCA, discriminant analysis therefore are employed herein to test if measurements are useful to identify unknown isolated teeth, assigning them to a specific taxon, and to infer phylogenetic hypotheses [51]. The significance (p-value) of each pairwise comparison was determined using Hotelling’s t^2^-test to determine significance at p < 0.05. Since the canonical variate analysis (CVA) can be considered an extension of discriminant analysis to more than two groups [32, 52] we used this multivariate approach to test hypotheses of morphologic similarities or differences in the overall sample among all groups. CVA projects a multivariate dataset down to two or more dimensions in a way that maximizes separation between three or more given groups [32].

### PCA and DA protocols

**Fig 1:**
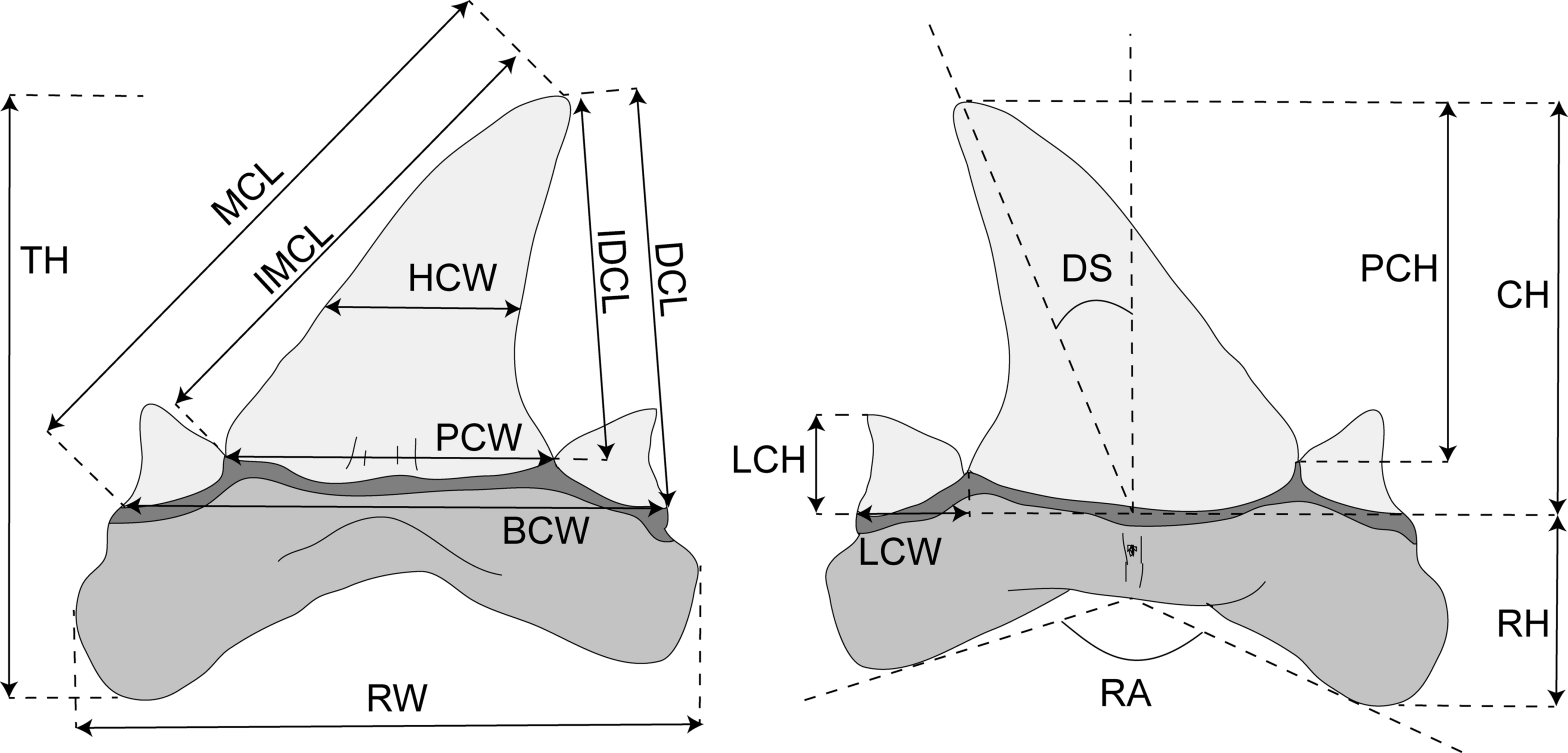
Morphometric tooth terminology. Abbreviations: BCW, basal crown width; CH, crown height; DCL, distal crown edge length; DS, degree of slant; IDCL, internal distal cutting edge length (cusp only); IMCL, internal mesial cutting edge length (cusp only); LCH, height of lateral cusplets; MCL, mesial crown edge length; PCH, height of principle cusp; PCW, width of principle cusp; RA, angle between root lobes; RH, root height; RW, root width; TH, total height of tooth.

A total of 14 linear measurements (in millimetres) and two angles (in degrees) were taken from images using the software package TPSdig 2.19 [53] following the scheme applied in some recent studies of fossil sharks [11, 24]. Measurements were taken to the nearest 0.01. Morphometric tooth terminology is adopted and modified from Kriwet *et al*. [24] to which we added new measurements that are useful also in case of incomplete specimens. The following measurements depicted in Fig 1 are evaluated here: basal crown width (BCW), crown height (CH), distal crown edge length (DCL), degree of slant (DS), half-crown width (HCW), inner distal crown edge length (IDCE), inner mesial crown edge length (IMCE), height of lateral cusplets (LCH), width of the lateral cusplets (LCW), mesial crown edge length (MCL), height of principle cusp (PCH), width of principle cusp (PCW), angle between root lobes (RA), root height (RH), root width (RW), total height of the tooth (TH). Morphological terminology mostly follows Cappetta [1].

Normal distribution of data is necessary in multivariate analyses [33]. For this reason raw data usually cannot be used. In fact, it is often assumed that biological and even more palaeontological data never follow a Gaussian distribution [54]. This is mainly due to the fact that in palaeontological analyses, as in our case study, some data might be missing and, on the other hand, it is very difficult to produce very large data sets that might render in some way data to be normally distributed. This problem can be solved using two simple procedures, which are also useful to minimize the variation caused by different sizes and ontogenies: 1) standardization and 2) log-transformation of data. Standardization of data consists in calculating the ratio between each trait and one of the measurements. In our study case we choose the total height of the tooth (TH) for standardization since this trait can be recognized in all our specimens. The choice of using TH (as sum of crown and root height) is also due to the fact that standardization eliminates this trait when it is divided by itself in all specimens (TH/TH = 1). Nevertheless, its morphological significance remains preserved since it is replaced by other traits (e.g. crown and root heights, since TH = CH + RH; see Fig 1). Standardization is useful to remove size effects so that shape will be the only trait analysable. In a recent study, Belben *et al*. [17] performed PCA based on fossil shark tooth measurements in order to investigate the ecological replacement in high trophic levels in marine settings. However, for their purposes, the authors did not standardize measurements so that the main morphological variation detected between Maastrichtian and Danian taxa was the size of the shark teeth, suggesting that post-extinction ecosystems were dominated by significantly smaller sharks [17]. The log-transformation of data is used here to overcome the problem of the non-normal distribution of data by un-stretching large scales of values [33, 55]. Moreover log-transformation is also useful to reduce considerably the variation due to ontogeny (allometric effect) since we assume that specimens of different developmental stages are employed here [56].

By employing two different measurements (millimetres and degrees) it is necessary to test differences in variation in using two different proxies [47, 57]. This can be done by calculating the coefficient of variation (COV, calculated as standard deviation divided by the trait mean) for each character (using size-corrected and log-transformed data) in order to obtain an estimate of trait variability. The differences between linear measurements and angles can be then tested using a non-parametric test as the Mann-Whitney U-test [47, 57].

The tooth shape of the various genera was studied by analysing the respective morphospaces detected by PCA performed on standardized and log-transformed data to obtain the principal component scores (PCs), the vectors describing the maximum variation of specimen shape. This enables to obtain direct visual images of the spatial separation of specimens. It is a common rule for multivariate statistical techniques to interpret only those components (usually the first two to four) that contribute more than 5% of the total variance, since the traits associated to other components explain only a minimum part of the variation [33, 58]. Finally, the component loading values of the main PCs can be used to interpret the 'meaning' of the components that is to identify the main factors to which an axis is related [59]. The higher (or the lower) the values, the stronger the correlation of the corresponding variable with the factor axes is.

In order to support the visual separation of the groups by PCA, significant differences can be tested using non-parametric tests. Since it is often assumed that standard statistics that directly compare abundance distribution (e.g. Chi-square) or parametric tests (e.g. ANOVA) may not be useful for non-normally distributed data we use here two non-parametric techniques, which do not require normal distributions. The multivariate analysis of variance (PERMANOVA) was applied to test similarities in-group centroid position between the different groups representing tooth position or taxa [60]. The analysis of similarities (ANOSIM) was employed to test quantitatively the degree of overlap between different groups [61]. ANOSIM measures how separate groups are, on a scale of 0 (indistinguishable) to 1 (all similarities within groups are less than any similarity between groups). The null hypotheses for PERMANOVA and ANOSIM are the similarity of the group centroids, and the equal medians and ranges for within-group ranked dissimilarities among groups, respectively [59]. Euclidean distances were chosen as distance measure for both tests and alpha was set at 0.05. All analyses were performed using the software package Paleontological Statistics PAST [59].

### Ethics statement

No permits were required for the described study, which complied with all relevant regulations.

## Results

Among the standardized and log-transformed morphometric measurements, the degree of slant shows the most variation (COV_DS_ = 1.1), followed by the root angle (COV_RA_ = 0.44). The least variable characters are the distal crown edge length (COV_DCL_ = 0.08) and crown height (COV_CH_ = 0.09). However, coefficients of variation between linear measurements and angles are not significantly different (Mann-Whitney U-test: mean ranks 6.9 and 1.1, respectively; p = 0.93) so they can be used together to perform multivariate analyses.

### PCA on the overall sample

**Fig 2:**
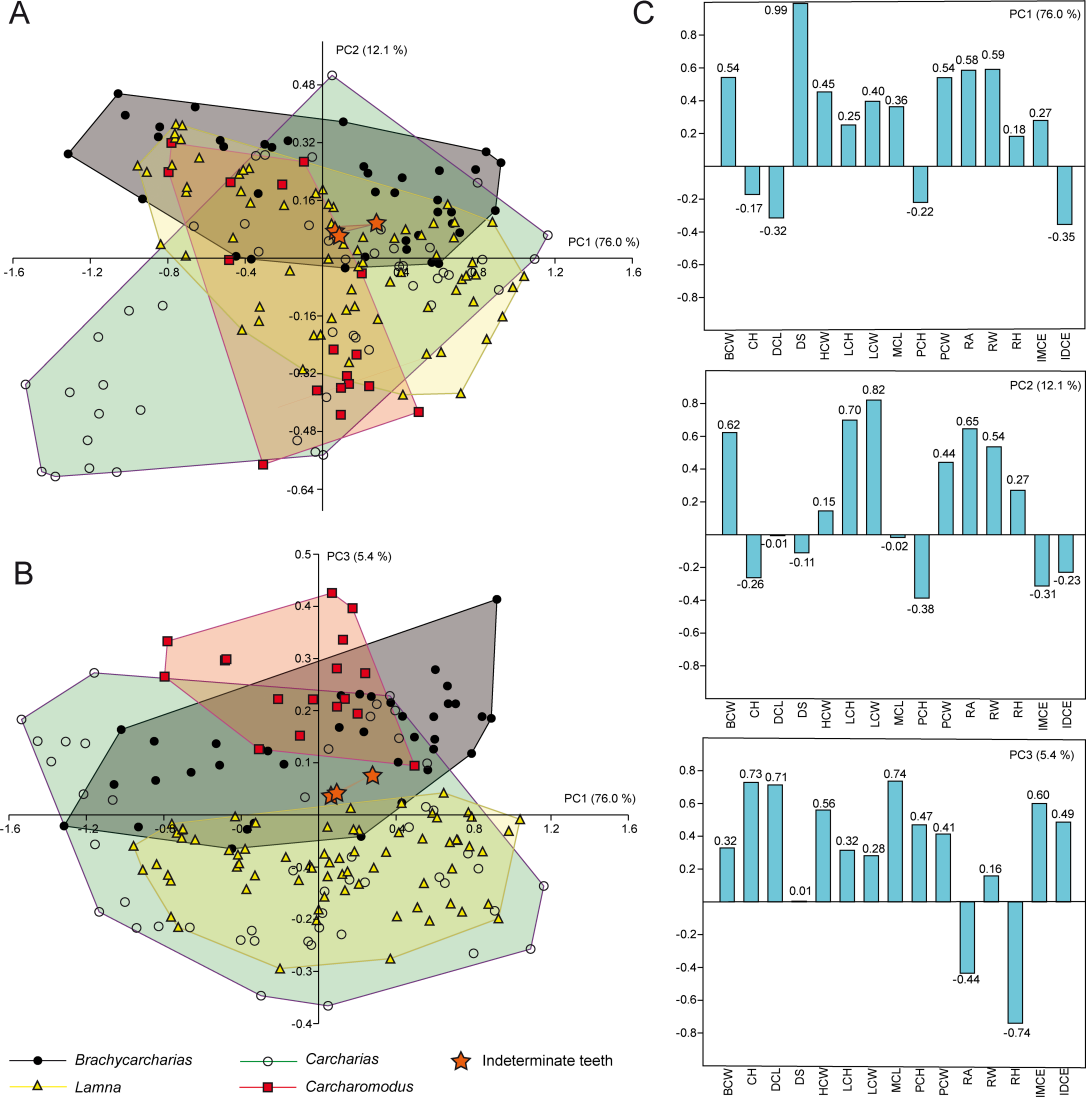
PCA. Results of PCA performed on the entire sample of standardized and log-transformed measurements for *Brachycarcharias*, *Lamna*, *Carcharias* and *Carcharomodus*, represented as convex hulls; (A) Morphospace plotted on PC1 and PC2; (B) Morphospace plotted on PC1 and PC3; (C) Loading values showing the variables associated with the first three PC axes.

The PCA performed on the entire sample including all the four genera and indeterminate specimens detected 15 PC axes, with the first three explaining more than 5% of variation and accounting together for 93.6% of the total variability. Eigenvalues and percentage of explained variability are represented in Table 1, whereas the morphospaces plotted on the first three axes and variables associated with each factor axis are shown in Fig 2.

**Table 1:** Principal component axes, eigenvalues and percent variation for the entire sample of 175 teeth.

PC	Eigenvalue	% variance	Associated variables
1	0.382950	76.036	DS
2	0.061111	12.134	LCH, LCW
3	0.027375	5.435	RH, CH, DCL, MCL
4	0.014626	2.904	
5	0.007384	1.466	
6	0.002935	0.583	
7	0.002568	0.510	
8	0.001563	0.310	
9	0.000865	0.172	
10	0.000830	0.165	
11	0.000608	0.121	
12	0.000546	0.108	
13	0.000158	0.031	
14	0.000098	0.020	
15	0.000025	0.005	

The marked monognathic heterodonty of lamniform teeth [1] contributes to the largest amount of variation in our data set. In particular, PC1 (76.0%) is mainly related to the degree of slant (DS). Positive values of PC1 are related to a strong inclination of the main cusp, a feature that mainly characterizes lateral teeth, whereas specimens with almost vertical cusps mostly representing anterior or antero-lateral teeth show negative values (0–1° DS). The PC2 (12.1%) is mainly related to the height and width of lateral cusplets (LCH and LCW). In particular, positive scores of PC2 are related to with specimens having tall and wide cusplets (e.g. *Brachycarcharias* is almost entirely confined in these values), whereas specimens having low and narrow cusplets (e.g. the anterior teeth of *Lamna* and the upper lateral teeth of *Carcharomodus*) are associated with mostly negative values. PC3 (5.4%) is mainly related to the ratio between root height (RH) and some measurements related to the height of the main cusp (CH, DCL and MCL). In its positive scores, specimens with very tall main cusp and low root are located (e.g. *Carcharomodus* is entirely confined in these scores), whereas negative ones are mainly related to lower crowns and higher roots (e.g. *Lamna nasus* specimens lie almost entirely in these values). The quantitative occupation patterns are supported by the results of non-parametric tests calculated along all PCs and for all possible pairwise comparisons (Table 2).

**Table 2:** PERMANOVA and ANOSIM. Nonparametric tests used to assess significant differences in morphospace occupation between the four genera. The significance is computed by permutation of group membership, with 9,999 replicates. Euclidean distances were chosen as a measure unit.

PERMANOVA				
**p-values**	*Brachycarcharias*	*Carcharias*	*Carcharomodus*	*Lamna*
Indeterminate	0.7051	0.493	0.061	0.477
*Brachycarcharias*		0.006*	0.005*	0.007*
*Carcharias*			0.046*	0.018*
*Carcharomodus*				0.004*
**F-values**				
Indeterminate	0.242	0.604	2.609	0.635
*Brachycarcharias*		6.694	6.450	6.491
*Carcharias*			3.392	4.740
*Carcharomodus*				7.158
ANOSIM				
**p-values**	*Brachycarcharias*	*Carcharias*	*Carcharomodus*	*Lamna*
Indeterminate	0.844	0.975	0.179	0.645
*Brachycarcharias*		0.004*	0.002*	0.0001*
*Carcharias*			0.081	0.0002*
*Carcharomodus*				0.0002*
**R-values**				
Indeterminate	-0.134	-0.203	0.158	-0.047
*Brachycarcharias*		0.087	0.197	0.163
*Carcharias*			0.080	0.117
*Carcharomodus*				0.224

* indicates significant comparisons (p < 0.05) thereby suggesting that groups exhibit considerably different morphospace occupation.

The overall p-values for PERMANOVA and ANOSIM are 0.0002 and 0.0001, respectively.

**Fig 3:**
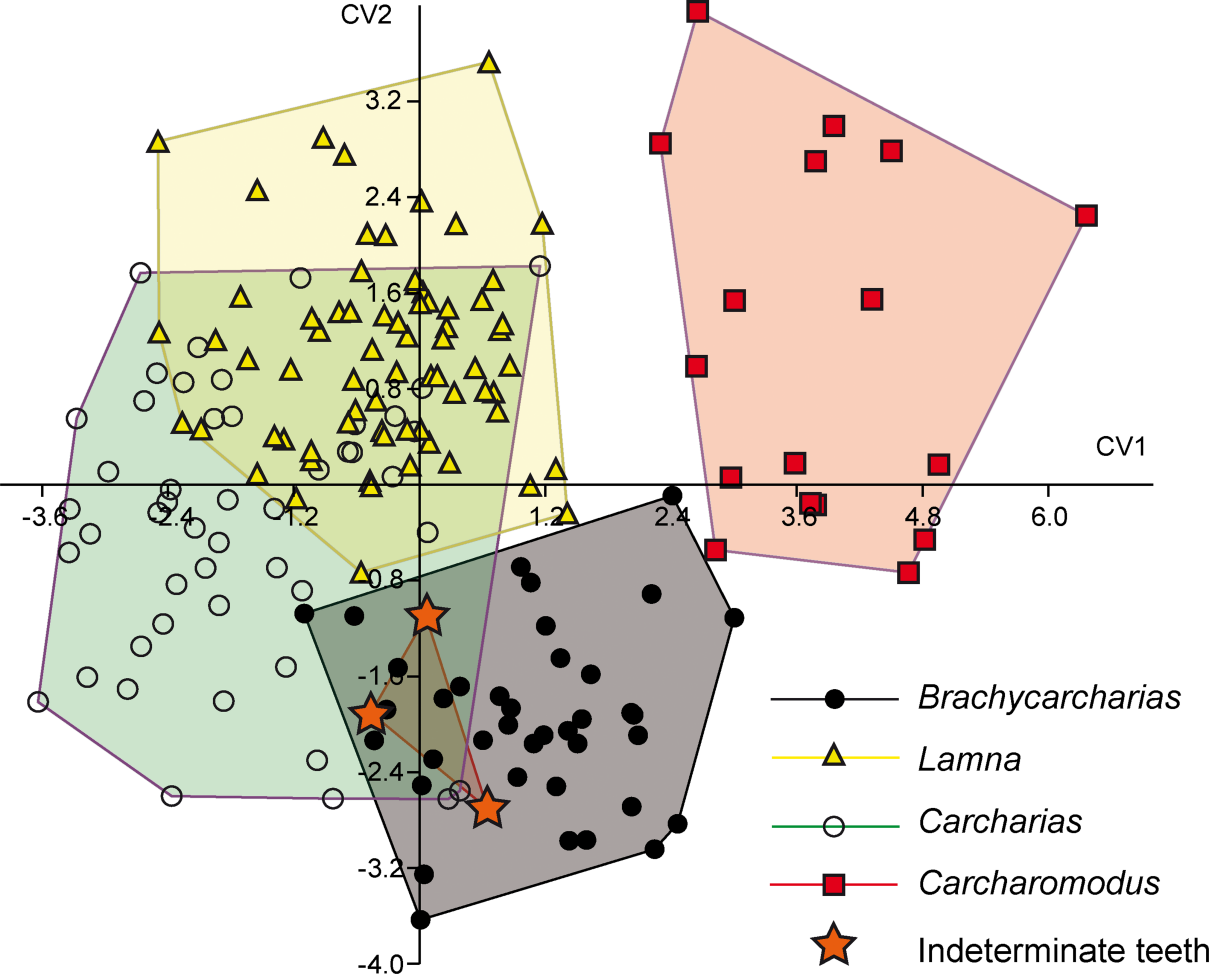
CVA. Results of CVA performed on the entire sample of standardized and log-transformed measurements. All genera are significantly separated (Hotelling’s p < 0.05) but the test failed to assign indeterminate teeth to a certain taxon (p > 0.05).

In general, both PERMANOVA and ANOSIM clearly support different morphospace occupations, suggesting that measurements are useful to separate genera. In particular, PERMANOVA rejects the null hypothesis of equal group centroids (p = 0.0005), whereas ANOSIM shows that convex hulls appear overlapped but are clearly different (R = 0.14; p = 0.0001). However, PCA is not useful to separate indeterminate teeth from other genera or assigning them to a certain taxon in the overall sample since all pairwise comparisons detected largely overlap with all convex hulls (PERMANOVA and ANOSIM: p > 0.05). Discriminant analysis of the overall sample detected through CVA (Fig 3 and Table 3) supports the hypothesis that the four known genera are clearly different (Hotelling’s p < 0.05). However, although CVA detected a significant separation of indeterminate teeth with those of *Lamna* (p < 0.05) the approach was not useful to assign them to *Brachycarcharias*, *Carcharias* or *Carcharomodus* (p > 0.05).

**Table 3:** CVA. Canonical variate analysis results of comparisons among *Brachycarcharias*, *Carcharias*, *Carcharomodus*, *Lamna* and indeterminate teeth.

Hotelling’s t test				
p-values	*Brachycarcharias*	*Carcharias*	*Carcharomodus*	*Lamna*
Indeterminate	0.911	0.264	0.538	0.045*
*Brachycarcharias*		> 0.0001*	> 0.0001*	> 0.0001*
*Carcharias*			> 0.0001*	> 0.0001*
*Carcharomodus*				> 0.0001*

* indicates significant comparisons (p < 0.05) obtained through Hotelling’s t test.

### *Brachycarcharias* vs *Lamna*

**Fig 4:**
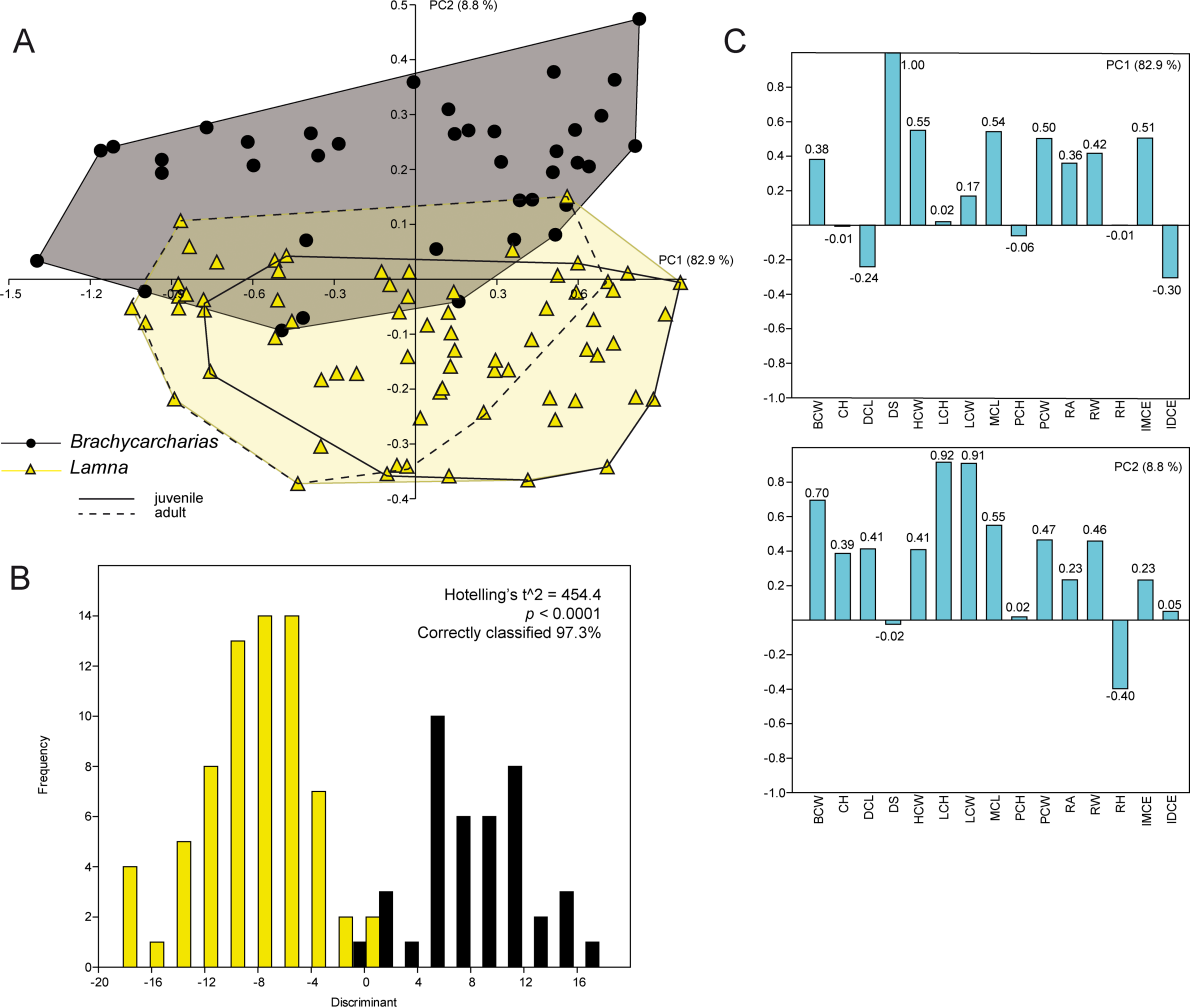
PCA. Results of the PCA performed on the standardized and log-transformed measurements for *Brachycarcharias* and *Lamna*. (A) Morphospace plotted on PC1 and PC2; (B) Discriminant analysis results; (C) Loading values showing the variables associated with the two PC axes.

Of the 15 axes produced by PCA for *B*. *lerichei* and *L*. *nasus*, only the first two explain more than 5% of the variation, accounting for 91.8% of the total variability. The PC1 (82.9%) is always related to the inclination of the cusp whereas PC2 (8.8%) is related to the height and width of the lateral cusplets (Fig 4). Although the different tooth positions appear to be distributed along the same values of the PC1 (anterior and lower teeth in negative values, upper teeth in positive values), the two genera appear clearly separated along the PC2, with little overlap. In particular, *Brachycarcharias* mostly occupies positive values associated with taller and wider lateral cusplets, whereas the *Lamna* morphospace lies in negative values linked to short and narrow cusplets. The significant separation of the two groups in the morphospace is clearly supported by PERMANOVA and ANOSIM (p = 0.0001). Pairwise discriminant analysis also shows that more than 97% of teeth are correctly assigned to their a priori groups, with the Hotelling’s t^2^-test suggesting a significant separation of the two morphotypes (p < 0.0001).

For this particular pairwise comparison, we used two sets of complete tooth series of *L*. *nasus* representing an adult and a juvenile developmental stage, in order to see if the small sized sand tiger shark *Brachycarcharias* [11] better overlaps the ecospace of one of the two ontogenetic stages of *L*. *nasus*. However, as shown is Fig 4, the two individuals of *Lamna* are almost entirely overlapping (although showing significant separation; ANOSIM and PERMANOVA: p < 0.05) and it seems there is more overlap of the convex hull of *Brachycarcharias* with that of the adult stage of *L*. *nasus* than with the convex hull of the juvenile individual.

### *Brachycarcharias* vs *Carcharias*

**Fig 5:**
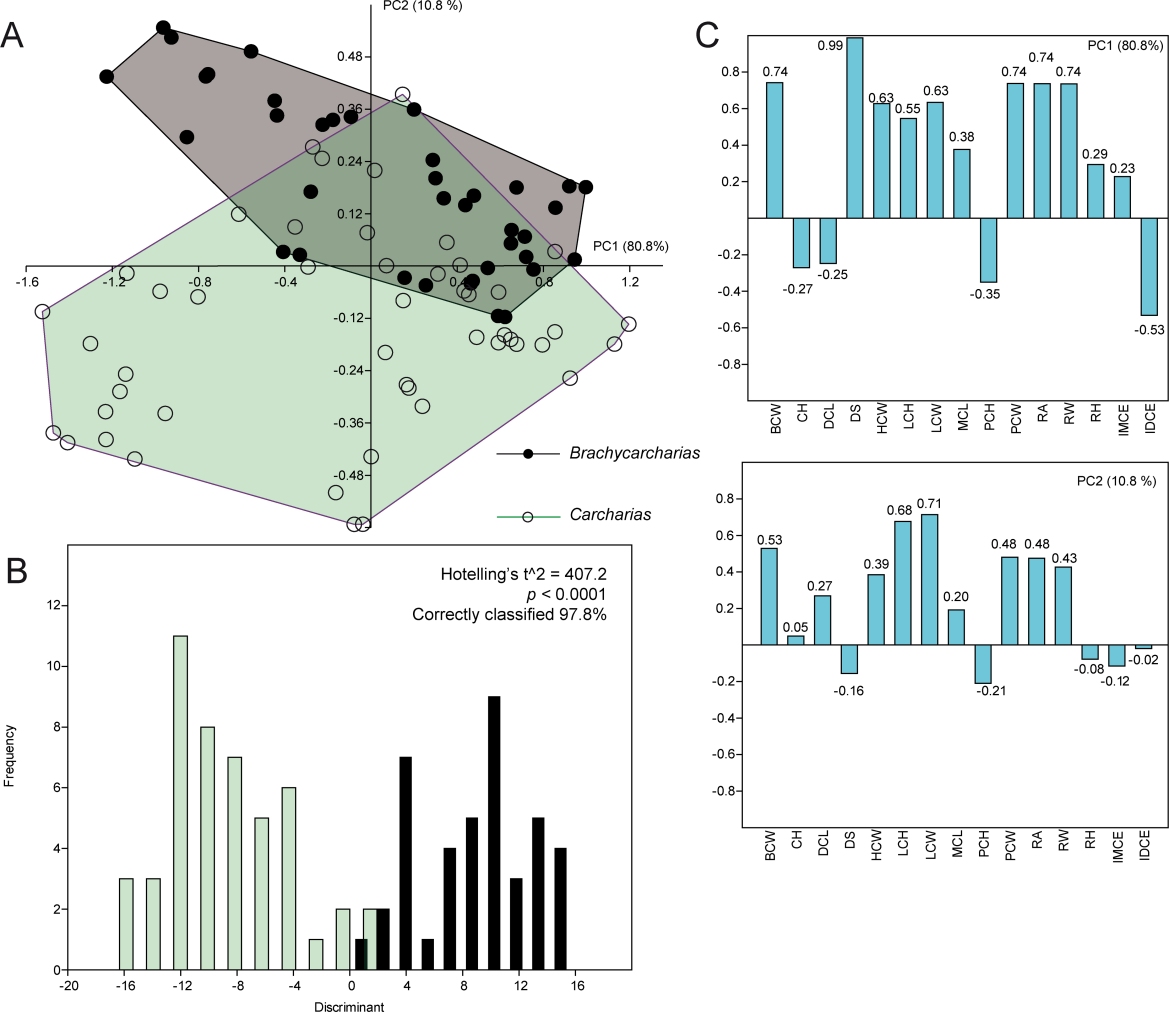
PCA. Results of the PCA performed on the standardized and log-transformed measurements for *Brachycarcharias* and *Carcharias*. (A) Morphospace plotted on PC1 and PC2; (B) Discriminant analysis results; (C) Loading values showing the variables associated with the two PC axes.

Only the first two PCs over 15 PCs explain more than 5% of the variation, accounting together for 91.6% of the total variability for this pairwise comparison. The main characters associated with PC1 (80.8%) and PC2 (10.8%) are the same as those in the previous comparison (Fig 5).

Although the different tooth positions appear to be distributed along the same values of the PC1 (anterior and lower teeth in negative values, upper teeth in positive values), the two genera are always separated along the PC2, with little overlap in positive values of PC1, suggesting that upper lateral teeth of *Brachycarcharias* and *Carcharias* have similar morphologies. However the main difference is related to the characters associated to anterior and antero-lateral teeth. In particular, these teeth in *Brachycarcharias* mostly occupy the quadrant with negative PC1 and positive PC2 values, since anterior and antero-lateral teeth of *Brachycarcharias* are characterized by tall lateral cusplets. On the contrary, anterior and antero-lateral teeth of *Carcharias* are located in the quadrant formed by negative values of both PCs, clearly associated with their very small cusplets. Significant separation of the two groups is supported by PERMANOVA and ANOSIM (p = 0.0001) and by discriminant analysis. This latter shows that 97.8% of the teeth are correctly assigned to their a priori groups, with the Hotelling’s t^2^-test suggesting a significant separation of the two morphotypes (p < 0.0001).

### *Brachycarcharias* vs *Carcharomodus*

**Fig 6:**
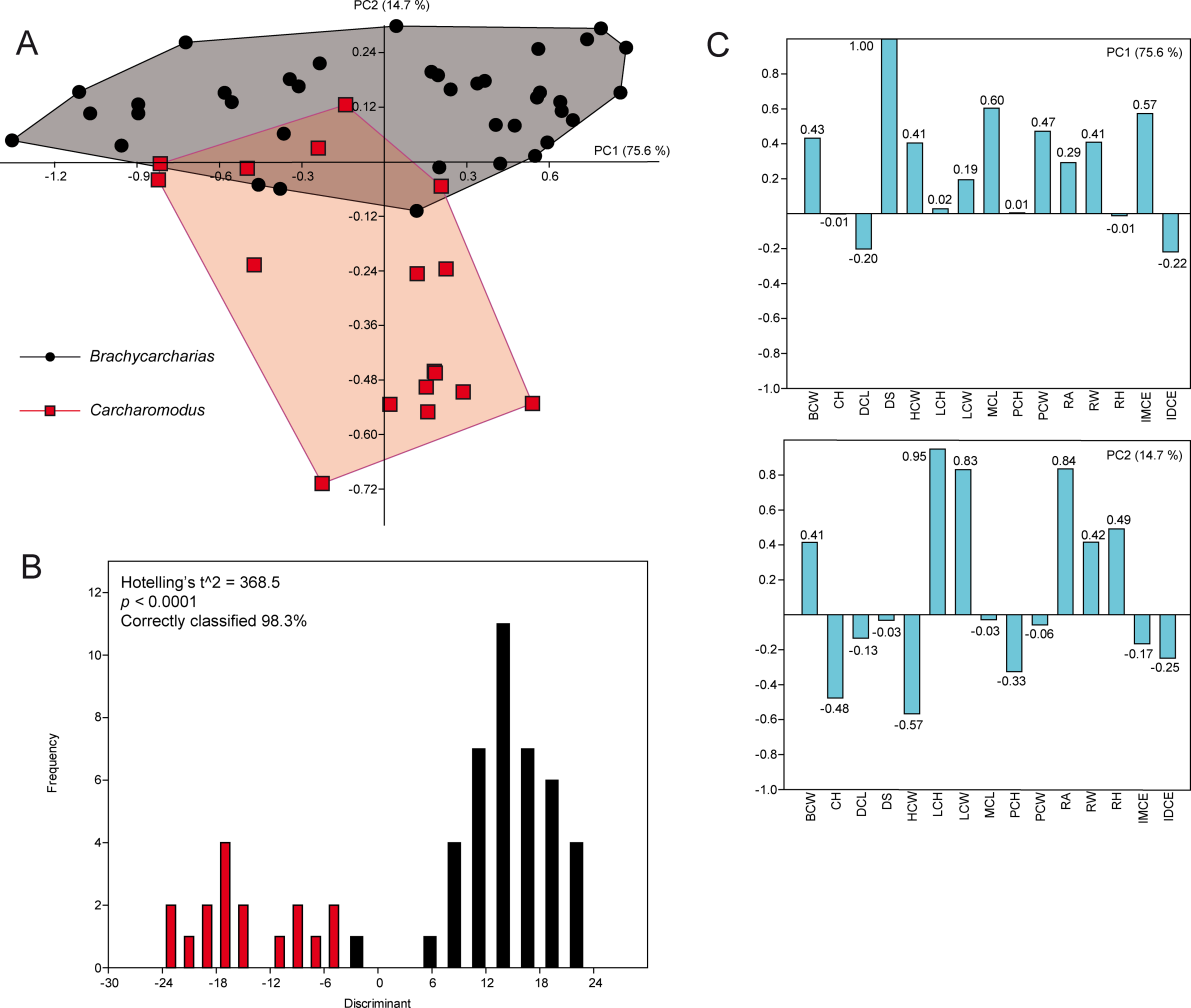
PCA. Results of the PCA performed on the standardized and log-transformed measurements for *Brachycarcharias* and *Carcharomodus*. (A) Morphospace plotted on PC1 and PC2; (B) Discriminant analysis results; (C) Loading values showing the variables associated with the two PC axes.

The PCA performed on the pairwise comparison between *Brachycarcharias* and *Carcharomodus* detected 15 PCs, with only the first two explaining more than 5% of variation and accounting together for 90.3% of the total variability. PC1 (75.6%) is always related to the degree of slant (DS), with positive values related to a strong distal inclination of the main cusp and negative scores related to teeth with an almost vertical cusp (Fig 6).

PC2 (14.7%) is related to the height and width of lateral cusplets (LCH and LCW), and in part also to the root angle (RA). Positive scores of PC2 are related to specimens having higher LCH, LCW and RA, whereas specimens having lower LCH, LCW and RA display negative values. Based on this, *Brachycarcharias* is thus confined in positive PC2 scores, whereas *Carcharomodus* lies along negative PC2 scores. Both PERMANOVA and ANOSIM clearly support different morphospace occupations, suggesting that measurements are useful to separate the two genera (p = 0.0001). Discriminant analysis shows that more than 98% of teeth are correctly assigned to their a priori groups, which are significantly separated (Hotelling’s t^2^-test: p < 0.0001).

### Identifying indeterminate shark teeth

**Fig 7:**
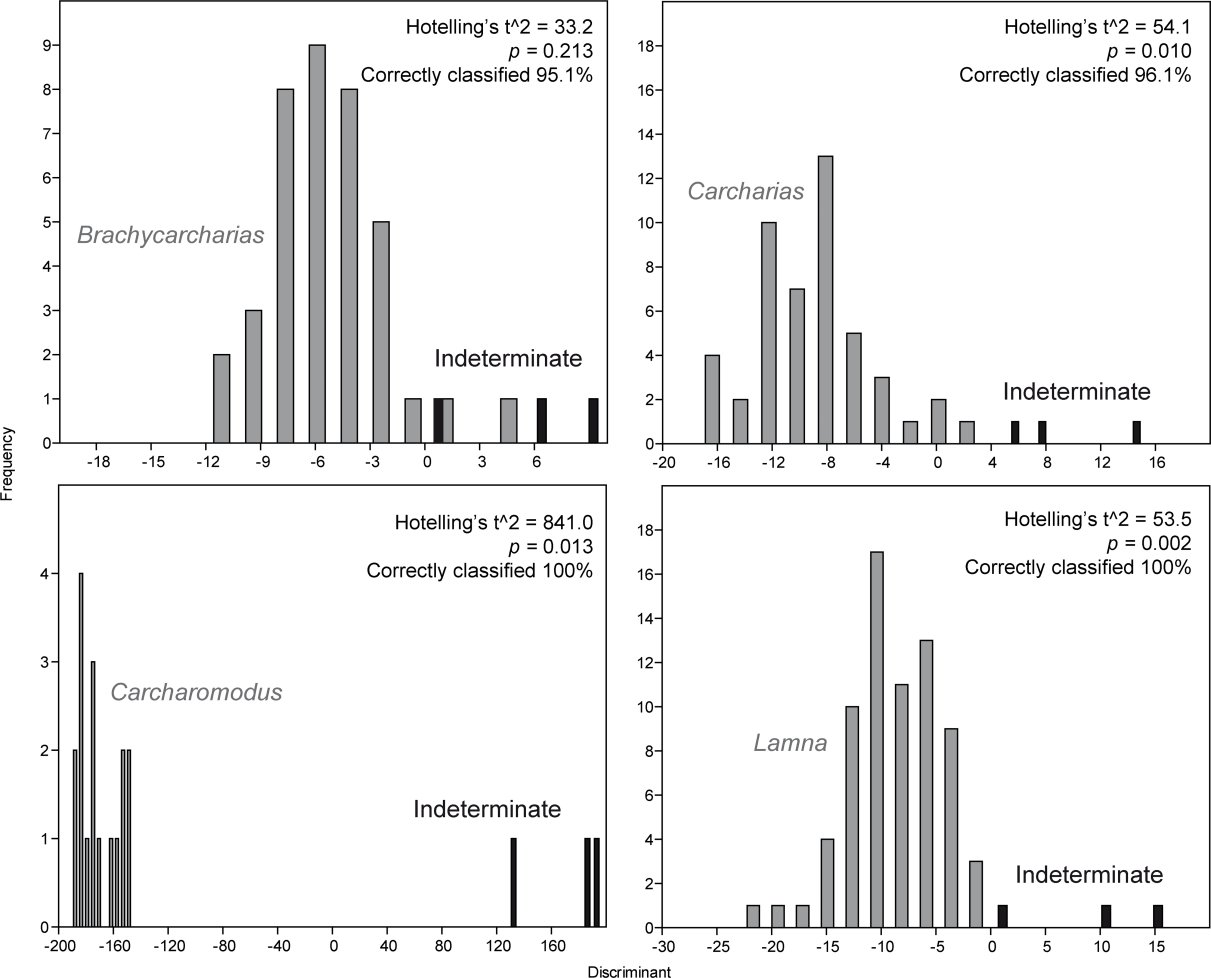
DA. Results of the discriminant analyses comparing indeterminate teeth with those of *Brachycarcharias*, *Lamna*, *Carcharomodus* and *Carcharias*.

Because the three indeterminate teeth show a large amount of overlap with all convex hulls of known genera (see Figs 2 and 3), PCA and CVA are not useful in this case to confidently determine, which taxon they belong to. However, despite the amount of overlap seen in PCA and CVA between indeterminate teeth and those of *Brachycarcharias*, *Lamna*, *Carcharomodus* and *Carcharias*, discriminant analyses performed as single pairwise comparisons (Fig 7) show that indeterminate teeth are significantly different from those of *Lamna*, *Carcharomodus*, and *Carcharias* (p < 0.05) whereas there is a good overlap and non-significant separation with those referred to *Brachycarcharias* (p > 0.05).

Discriminant analysis and associated Hotelling’s t^2^-test therefore suggest that unidentified teeth can be confidently assigned to *Brachycarcharias* based on non-significant differences between these two samples (p > 0.05) and on significant differences between indeterminate teeth and those referred to *Carcharias*, *Carcharomodus* and *Lamna* (p < 0.05). This suggests that although the indeterminate teeth are morphologically similar to some of the teeth of the known genera, minimal differences in their proportions can be successfully detected by pairwise discriminant analyses.

## Discussion

### Traditional morphometrics as tool for inferring taxonomy and phylogeny

The primary goal of this paper was to demonstrate that a quantitative approach based on traditional morphometrics is very useful to support taxon identifications. For this purpose, as case study, we intend to finally solve a recent taxonomic debate, which involved the validity of the extinct sand tiger shark *Brachycarcharias*, because Purdy & Francis [10] challenged the validity of this taxon stating that there are no robust morphological evidences to create a new genus for the species *Lamna lerichei* Casier, 1946. Furthermore, although Maisch *et al*. [12] considered *Brachycarcharias* as valid taxon, the authors suggested that it might have had similar feeding and habitat preferences as the living porbeagle shark, *Lamna nasus*, because of similar tooth morphologies. On the contrary, our analysis detected significant differences in tooth morphologies between *Brachycarcharias* and *Lamna*, mainly due to different proportions of the lateral cusplets with respect to the main crown. These differences in morphology also support the interpretations of Marramà *et al*. [11] that *L*. *nasus* is a pelagic or epipelagic lamnid shark that is known to inhabit coastal temperate to cool waters on continental shelves, but also occurs far offshore in ocean basins but occasionally also close inshore, from the North Atlantic to temperate waters of the Southern Hemisphere. However, so far this species has never been found in equatorial tropical seas [62–64]. Conversely, teeth of *Brachycarcharias* were abundantly recovered from tropical shallow to cooler deep-water deposits distributed worldwide [11]. It is therefore most likely that *Brachycarcharias* was an opportunistic Palaeogene top predator with a wide range of feeding and habitat preferences contrary to the assumptions of Maisch *et al*. [12]. Moreover, Marramà *et al*. [11] also demonstrated that PCA is additionally useful, at least partially, to distinguish the teeth from different jaw positions in *Brachycarcharias lerichei*. This indicates that PCA and associated non-parametric tests actually represents a powerful tool for identifying not only different taxa at least at genus level but also tooth position within jaws.

Although PCA results in apparent overlaps of morphotypes, discriminant analysis might be extremely useful to support the classification of isolated indeterminate teeth through pairwise comparisons. In fact, the combination of qualitative dental character of the three isolated teeth collected from the Ypresian (Early Eocene) deposit of the La Meseta Formation on Seymour Island, Antarctica (e.g. teeth up to 25 mm with fairly low triangular cusp decreasing regularly in width; one to two pairs of well-developed lateral cusplets; root with broadly separated lobes; upper teeth with a cusp bent distally [1, 25]) confirm the results of the discriminant analysis and support their assignment to the extinct genus *Brachycarcharias* Cappetta & Nolf, 2005.

**Fig 8:**
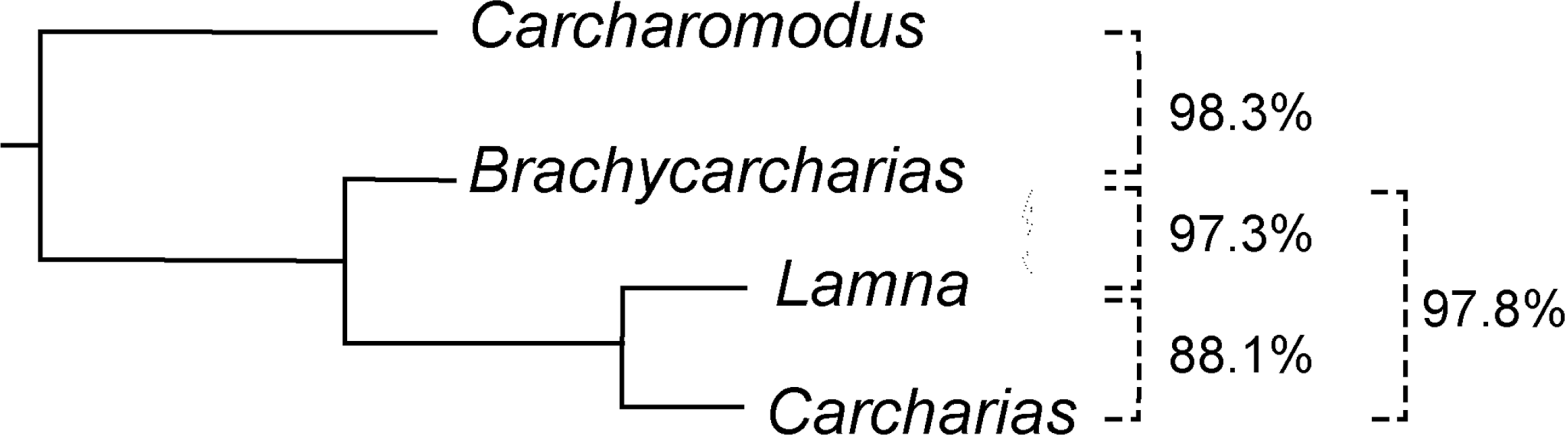
Hierarchical classification tree. Hypothetical relationships between *Carcharomodus*, *Brachycarcharias*, *Carcharias* and *Lamna* based on the percentage of teeth correctly classified by discriminant analysis performed as pairwise comparisons. All the other possible combinations (*Carcharomodus*/*Lamna*; *Carcharomodus*/*Carcharias*) are related to a 100% of teeth correctly classified.

The degree of separation among tooth clusters detected by discriminant analysis can be used as tool to reveal taxonomic entities [65] and also phylogenetic signals [51, 66, 67]. In our case study presented here (Fig 8) all species are unambiguously identified and supported by our analyses. The large similarities of teeth of *Lamna* with teeth of *Carcharias* rather than with teeth of *Brachycarcharias* and even more than with those of *Carcharomodus*, however, might also include a functional signal in tooth morphology, since *Carcharomodus* is supposed to be closely related to the extant white shark, *Carcharodon carcharias* [24] and closer relationships to *Lamna* thus would have been expected in our analysis. Consequently, the position of *Carcharomodus* at the basis of the hierarchical classification tree (Fig 8) might indicate that the signal here is functional rather than phylogenetic.

Consequently, the presence of a phylogenetic signal remains ambiguous because the taxonomic arrangement in the classification tree is not consistent with their placement on phylogenetic trees. Nevertheless, the hypothetical relationships detected through the discriminant analysis might support differences in recent studies based on morphological and molecular data, in which Odontaspididae are revealed to be non-monophyletic [65–71] conversely to the traditional view that both, *Carcharias* and *Odontaspis*, are distinct from all other lamniforms using specific sets of characters [1, 72, 73]. The results thus would indicate that the Palaeogene *Brachycarcharias* represents the sister taxon to a monophyletic group formed by Odontaspidae and Lamnidae rather than being a genuine member of sand tiger sharks as traditionally assumed [1, 25]. Nonetheless, this interpretation needs to be verified by inclusion of additional lamniform taxa because the support (distances between taxa in the classification tree) is weak. Therefore, we do not exclude the possibility that the similar morphologies might be the result, at least in part, of convergent and/or parallel evolutionary processes.

### Traditional vs geometric morphometrics

Traditional morphometrics is often underestimated or discarded for quantitative analyses in paleobiology mostly because of the several cons that this approach shows with respect to geometric morphometrics (e.g. measurements are highly related to size and contain little information about the shape contained in an object, it is not possible to reconstruct graphical representations of the shape, measurements taken from two different shapes can produce equal results [58]). However, some of these problems can be overcome. Although measurements are highly related to size, and contain little information about the shape contained in an object, their standardization and log-transformation eliminate the size-effect, so that the only parameter detected by the PCA will be the differences in shape. Moreover, taking a high number of measurements and producing a large data set can be useful to overcome the problem of the non-normality in data distribution.

We demonstrated that PCA and DA are useful methods to detect minimal differences in tooth morphologies in some selected taxa of lamniform sharks. Although a few quantitative studies on isolated fossil teeth were performed, they were mainly based on geometric morphometric approaches [15, 16]. The landmark-based approach is certainly a powerful tool to explore geometric differences between biological forms and to detect patterns of morphospace occupation related to changes in shape [58, 74, 75]. In the last years several authors have successfully used this approach in palaeontology to avoid taxon over-splitting, to detect morphospace occupation and diversification through time, and to identify morphological adaptations and evolutionary convergences, among others [22, 23, 76–80]. However, geometric morphometrics sometimes might not be the most reliable choice in palaeontological analyses. The main reason is that the landmark-based approach is extremely sensitive to deformation due to extrinsic factors as taphonomic distortions [78]. Another problem is that geometric morphometrics is intolerant of missing data, which can preclude the analysis of poorly preserved or damaged specimens [81–84], reducing inclusion of fossilized specimens. The main limits working with isolated fossil shark teeth is that they are often preserved damaged and therefore incomplete, so that: 1) the number of landmarks can be insufficient to capture the whole shape, if homologous points recognizable in all specimens have to be selected; 2) choosing a good number of landmarks on a small well-preserved subsample might influence the results since the sample might be not significantly large enough to perform statistical tests. It is mandatory to use homologous landmarks or semilandmarks in all specimens examined in geometric morphometrics [58]. Incomplete specimens, lacking parts where a determinate landmark is impossible to be imputed, cannot be used. This might be solved by eliminating a certain landmark from the analysis, but with consequent loss of information.

Traditional morphometrics can be therefore a good alternative to geometric morphometrics when working with incomplete specimens as fossil shark teeth, which do not allow for recognizing a significant number of homologous points. In fact, PCA replaces missing data using pairwise substitution allowing the inclusion of partially incomplete specimens in the sample [59]. This may allow building a larger and more robust data set, therefore enhancing the reliability of the sample.

## Conclusions

A recent controversy about the validity of the extinct sand tiger shark *Brachycarcharias* was the initial trigger for analysing, through rigorous multivariate approaches and statistical tests, whether traditional morphometrics may represent a reliable approach in supporting the taxonomic identification of isolated fossil shark teeth. The large sample of isolated teeth of four living and fossil lamniform genera used here provide the opportunity for investigating the intergeneric variability in tooth morphologies but also in relation to their jaw positions. The multivariate analyses revealed that the morphometric approach using linear and angular measurements is able to detect significant differences among different taxa, therefore supporting the taxonomic identification based on qualitative characters. Moreover, we demonstrated that discriminant analyses are particularly useful to assign a set of indeterminate teeth to a certain taxon through single pairwise comparisons. Finally, this approach provides also opportunities to further investigate possible functional and/or phylogenetic signals in fossil shark teeth as shown from the discriminant analysis comparisons of *Brachycarcharias*, *Lamna*, *Carcharias*, and *Carcharomodus* in this study. However, it must be pointed out that this approach does not replace qualitative analyses in any way, but complements qualitative approaches and provides additional support for identifications.

## Supporting information

S1 Table List of the material used in the study and related tooth measurements. (DOCX)
