# Supplementary material for: Principal component and discriminant analyses as powerful tools to support taxonomic identification and their use for functional and phylogenetic signal detection of isolated fossil shark teeth
Source: PLoS One. 2017 Nov 28;12(11):e0188806. doi: 10.1371/journal.pone.0188806 (PMC5705141; doi:10.1371/journal.pone.0188806)
Supplement: S1 Table — (DOCX) [file pone.0188806.s001.docx]

**S1 Table.** List of the material used in the study and related tooth measurements (lengths in mm; angles in degree). Abbreviations: BCW, basal crown width; CH, crown height; DCL, distal crown edge length; DS, degree of slant; IDCL, internal distal cutting edge length (cusp only); IMCL, internal mesial cutting edge length (cusp only); LCH, height of lateral cusplets; MCL, mesial crown edge length; PCH, height of principle cusp; PCW, width of principle cusp; RA, angle between root lobes; RH, root height; RW, root width; TH, total height of tooth. For recent taxa (*Lamna nasus* and *Carcharias taurus*), since the position of each tooth is known, the relative position of each single tooth is identified by the following scheme: U/L (upper/lower jaw) + A/L (anterior/lateral position); + number of relative position + L/R (left/right side of jaw). Institutional abbreviations: MC, Massimo Cerato’s registered collection, Italy; MCSNV, Museo Civico di Storia Naturale di Verona; MGP-PD, Museo di Geologia e Paleontologia dell’Università degli Studi di Padova; MNU, Museum für Natur und Umwelt, Lübeck; NHMUK, Natural History Museum of London; NHMW, Naturhistorisches Museum of Vienna; NRM-PZ, Swedish Natural History Museum, Stockholm; SMNK, State Museum of Natural History, Karlsruhe; SMNS, State Museum of Natural History Stuttgart; ZC UniVie, Zoological Collection, Department of Palaeontology, University of Vienna.

| **Taxon** | **Repository n.** | **BCW** | **CH** | **DCL** | **DS** | **HCW** | **LCH** | **LCW** | **MCL** | **PCH** | **PCW** | **RA** | **RW** | **RH** | **TH** | **IMCE** | **IDCE** | **Position** |
| --- | --- | --- | --- | --- | --- | --- | --- | --- | --- | --- | --- | --- | --- | --- | --- | --- | --- | --- |
| *Brachycarcharias lerichei* | NRM-PZ P15772 | 11 | 9.71 | 10.56 | 8.14 | 2.96 | 2.8 | 1.81 | 11.94 | 8.56 | 6.91 | 121.71 | 11.72 | 3.43 | 13.14 | 9.88 | 8.8 | upper antero-lateral |
| *Brachycarcharias lerichei* | NRM-PZ P15773 | 10.42 | 9.41 | 9.94 | 13.67 | 2.93 | 2.26 | 2.21 | 11.82 | 8.56 | 6.31 | 122.66 | 11.72 | 3.6 | 13.01 | 10.01 | 8.58 | upper lateral |
| *Brachycarcharias lerichei* | NRM-PZ P15779 | 9.42 | 9.15 | 10.28 | 1.8 | 3.06 | 2.17 | 1.93 | 10.29 | 8.11 | 5.69 | 117.15 | 11.56 | 3.76 | 12.91 | 8.64 | 8.52 | lower antero-lateral |
| *Brachycarcharias lerichei* | NRM-PZ P15836 | 7.97 | 8.14 | 9.19 | 1.8 | 2.32 | 1.97 | 1.53 | 8.87 | 7.23 | 4.57 | 123.68 | 9.6 | 2.72 | 10.86 | 7.48 | 7.72 | upper antero-lateral |
| *Brachycarcharias lerichei* | NRM-PZ P15926 | - | 8.88 | 9.97 | 1 | 2.45 | 1.92 | 1.85 | - | 7.76 | 5.64 | - | - | 3.53 | 12.41 | 8.83 | 8.13 | lower antero-lateral |
| *Brachycarcharias lerichei* | NRM-PZ P15839 | - | 9.56 | - | 0.5 | 2.86 | 2.35 | 1.55 | 10.61 | 8.57 | 6.19 | - | - | 3.97 | 13.53 | 9.1 | 9.03 | lower antero-lateral |
| *Brachycarcharias lerichei* | NRM-PZ P15841 | 10.58 | 7.21 | 7.89 | 16.69 | 2.63 | 1.83 | 2.28 | 10.2 | 6.31 | 6.55 | 138 | 11.42 | 3.21 | 10.42 | 8.37 | 6.39 | upper lateralmost |
| *Brachycarcharias lerichei* | NRM- PZ P15845 | - | 8.56 | 9.32 | 1.65 | 2.33 | 1.75 | 1.54 | - | 7.77 | 4.75 | - | - | 4.16 | 12.72 | 8.2 | 8.1 | lower antero-lateral |
| *Brachycarcharias lerichei* | NRM- PZ P15847 | - | 9.76 | 10.69 | 0.3 | 2.59 | 2.02 | 1.49 | - | 8.51 | 6.16 | - | - | 2.62 | 12.38 | 9.11 | 9.09 | lower antero-lateral |
| *Brachycarcharias lerichei* | NRM-PZ P15871 | - | 9.57 | 8.36 | 0.3 | - | 2.25 | 1.93 | - | 7.96 | - | - | - | 4.3 | 13.87 | - | 10.5 | lower antero-lateral |
| *Brachycarcharias lerichei* | NRM-PZ P15876 | 10.18 | 9.63 | 10.42 | 5.05 | 2.74 | 2.28 | 2 | 11.4 | 8.44 | 6.34 | 130.33 | - | 2.6 | 12.23 | 9.53 | 8.72 | upper antero-lateral |
| *Brachycarcharias lerichei* | NRM-PZ P15859 | 8.98 | 9.58 | 10.52 | 1 | 2.78 | 2.52 | 1.71 | 10.57 | 8.47 | 5.23 | - | - | 3.39 | 12.97 | 8.9 | 8.83 | lower antero-lateral |
| *Brachycarcharias lerichei* | NRM-PZ P15870 | - | 10.64 | 11.5 | 0.5 | 2.53 | 2.23 | 1.6 | 11.5 | 9.41 | 5.94 | - | - | 2.82 | 13.46 | 9.53 | 9.51 | lower antero-lateral |
| *Brachycarcharias lerichei* | NRM-PZ P15848 | 11.52 | 10.2 | 10.57 | 16.69 | 2.9 | 2.02 | 2.19 | 13.28 | 9.39 | 6.96 | 132.27 | 14.23 | 3.26 | 13.46 | 11.01 | 9.16 | upper lateral |
| *Brachycarcharias lerichei* | NRM-PZ P15872 | 12.3 | 12.02 | 12.2 | 18.52 | 3.72 | 2.76 | 2.08 | 15.39 | 10.21 | 8.1 | - | - | 5.39 | 17.41 | 12.67 | 10.37 | upper lateral |
| *Brachycarcharias lerichei* | NRM-PZ P15873 | - | 8.9 | 10.12 | 1.65 | 2.55 | 2.16 | 2.25 | - | 8 | 5.72 | - | - | 3.71 | 12.61 | 8.59 | 8.41 | lower antero-lateral |
| *Brachycarcharias lerichei* | NRM-PZ P15874 | 9.41 | 8.4 | 9.23 | 11.7 | 2.97 | 1.8 | 1.77 | 10.29 | 7.86 | 6.74 | 142.48 | 11.5 | 3.42 | 11.82 | 9.67 | 8.07 | upper lateral |
| *Brachycarcharias lerichei* | NRM-PZ P15806 | 8.93 | 7.42 | 8.38 | 6.64 | 2.41 | 1.61 | 1.57 | 8.99 | 6.66 | 5.85 | 130.69 | - | 2.94 | 10.36 | 7.57 | 7.08 | upper lateral |
| *Brachycarcharias lerichei* | NRM-PZ P15885 | - | 8.6 | - | 4.99 | 2.79 | 2.13 | 1.77 | 10.99 | 7.37 | 6.9 | 128.68 | - | 2.94 | 11.54 | 8.9 | 7.63 | upper lateral |
| *Brachycarcharias lerichei* | NRM-PZ P15796 | 11.47 | 10.98 | 11.62 | 13.4 | 3.36 | 2.5 | 2.09 | 13.44 | 9.99 | 7.2 | 133.61 | 15.43 | 4.43 | 15.41 | 11.75 | 10.42 | upper lateral |
| *Brachycarcharias lerichei* | NRM-PZ P15882 | - | 11.47 | - | 11.72 | 3.89 | 2.41 | 1.92 | 14.08 | 9.89 | 8.59 | - | - | 3.81 | 15.28 | 11.86 | 10.09 | upper lateral |
| *Brachycarcharias lerichei* | NRM-PZ P15857 | 11.3 | 9.52 | 10.03 | 12.55 | 3.24 | 2.9 | 2.23 | 12.47 | 8.22 | 6.87 | 130.54 | 12.86 | 3.15 | 12.67 | 10.03 | 8.21 | upper lateral |
| *Brachycarcharias lerichei* | NRM-PZ P15886 | 8.41 | 6.22 | 6.74 | 12.68 | 2.11 | 1.65 | 1.39 | 8.56 | 5.44 | 5.15 | 135.56 | - | 2.47 | 8.69 | 7.03 | 5.62 | upper lateral |
| *Brachycarcharias lerichei* | NRM-PZ P15888 | 9.52 | 8.14 | 9.12 | 3.43 | 2.62 | 2.48 | 1.89 | 9.84 | 6.98 | 5.61 | 125.45 | 12.26 | 3.18 | 11.32 | 7.74 | 7.33 | lower lateral |
| *Brachycarcharias lerichei* | NRM-PZ P15840 | - | 10.23 | 10.59 | 16.2 | 2.7 | 2.37 | 1.82 | - | 9.29 | 6.7 | - | - | 3.56 | 13.79 | 10.81 | 9.39 | upper lateral |
| *Brachycarcharias lerichei* | NRM-PZ P15777 | 7.74 | 9 | 9.74 | 0.66 | 2.26 | 2.55 | 1.62 | 9.8 | 7.94 | 4.73 | 123.87 | 9.95 | 3.04 | 12.04 | 8.27 | 8.25 | lower antero-lateral |
| *Brachycarcharias lerichei* | NRM-PZ P15887 | - | 9.25 | - | 13.2 | 3.18 | 1.98 | 1.65 | 11.64 | 8.18 | 7.46 | 147.44 | - | 1.97 | 11.22 | 9.88 | 7.92 | upper lateral |
| *Brachycarcharias lerichei* | MC 89 | 10.63 | 10.88 | 11.62 | 6.11 | - | 3.01 | - | 12.94 | 9.28 | 5.63 | 120.97 | 12.93 | 4.38 | 15.26 | - | - | upper antero-lateral |
| *Brachycarcharias lerichei* | MCSNV IG.135777/8 | 8.02 | 12.75 | 13.28 | 1.02 | - | 2.56 | - | 13.33 | 11.71 | 5.22 | 101.1 | 10.68 | 5.81 | 18.56 | - | - | lower antero-lateral |
| *Brachycarcharias lerichei* | MCSNV IG.135779 | 10.05 | 13.32 | 13.81 | 1.01 | - | 2.31 | - | 14.69 | 10.83 | 5.86 | 102.42 | 11.61 | 4.87 | 18.19 | - | - | lower antero-lateral |
| *Brachycarcharias lerichei* | MCSNV IG.23598 | 10.29 | 8.66 | 9.15 | 22.52 | - | 2.25 | - | 11.74 | 8.23 | 6.71 | 126.25 | 13.87 | 4.9 | 13.56 | - | - | upper lateral |
| *Brachycarcharias lerichei* | MCSNV IG.VR.24423 | 7.48 | 11.26 | 11.81 | 1.09 | - | 3 | - | 12.41 | 9.32 | 3.43 | 106.31 | 11.16 | 4.51 | 15.77 | - | - | anterior |
| *Brachycarcharias lerichei* | MCSNV IG.VR.69484 | 10.94 | 10.16 | 10.46 | 16.67 | - | 2.49 | - | 13.43 | 9.22 | 7.17 | 131.04 | 12.58 | 4.07 | 14.23 | - | - | upper lateral |
| *Brachycarcharias lerichei* | MCSNV IG.VR.69800 | 5.54 | 9.2 | 9.57 | 1.1 | - | 2.4 | - | 9.56 | 7.81 | 3.47 | 86.03 | 6.4 | 2.89 | 12.09 | - | - | anterior |
| *Brachycarcharias lerichei* | MCSNV T.176 | 14.4 | 9.38 | 9.85 | 27.19 | - | 2.88 | - | 14.99 | 8.11 | 8.41 | 120.15 | 16.82 | 4.91 | 14.29 | - | - | upper lateral |
| *Brachycarcharias lerichei* | MGP-PD 7366 | 12.85 | 14.22 | 15.29 | 5.36 | - | 3.47 | - | 16.09 | 12.48 | 8.68 | 125.01 | 15.93 | 5.3 | 19.52 | - | - | upper antero-lateral |
| *Brachycarcharias lerichei* | NHMUK PV.OR.43450 | 7.18 | 10.52 | 10.79 | 1.1 | - | 2.89 | - | 11.5 | 9.5 | 4.71 | 103.99 | 9.42 | 4.61 | 15.13 | - | - | anterior |
| *Carcharias acutissima* | SMNS 80739.74 | 11.49 | 11.94 | 12.52 | 8.81 | 3.8 | 2.87 | 1.89 | 14.25 | 10.54 | 7.27 | 133.18 | 15.05 | 3.9 | 15.84 | 13.2 | 12.11 | upper lateral |
| *Carcharias acutissima* | SMNS 87457.312 | 10.42 | 12.18 | 12.45 | 11.21 | 3.42 | 2.99 | 1.38 | 14.69 | 10.7 | 7.36 | 130.15 | 13.32 | 3.2 | 15.38 | 12.28 | 10.06 | upper lateral |
| *Carcharias acutissima* | SMNS 87457.321 | 11.34 | 12.7 | 13.42 | 9.46 | 2.96 | 3 | 2.13 | 14.79 | 11.56 | 7.62 | 116.72 | 14.51 | 4.28 | 16.98 | 13.19 | 11.84 | upper lateral |
| *Carcharias acutissima* | SMNS 87457.322 | 7.58 | 13.9 | 14.39 | 0.98 | 2.82 | 1.07 | 0.92 | 14.59 | 13.32 | 5.41 | 82.28 | 11.18 | 6.57 | 20.47 | 13.37 | 13.11 | anterior |
| *Carcharias acutissima* | SMNS 87457.324 | 12.32 | 5.33 | 7.76 | 0.5 | 3.44 | 1.86 | 2.24 | 8.73 | 4.4 | 7.47 | 116.06 | 16.16 | 5.01 | 10.34 | 6.22 | 5.98 | lateralmost |
| *Carcharias cuspidata* | SMNS 80740.13 | 11.5 | 27.71 | 27.65 | 1.5 | 5.39 | 1.93 | 1.69 | 29.49 | 25.31 | 10.13 | 82.77 | 13.86 | 8.4 | 36.11 | 28.8 | 26.44 | anterior |
| *Carcharias cuspidata* | SMNS 87457.301 | 12.26 | 27.31 | 27.86 | 1 | 6.26 | 2.27 | 1.45 | 28.53 | 26.27 | 9.98 | 86.83 | 18.58 | 8.8 | 36.11 | 27.71 | 26.73 | anterior |
| *Carcharias cuspidata* | SMNS 87457.302 | 19.9 | 41.6 | 42.52 | 1.02 | 9.55 | 3.72 | 2.23 | 43.34 | 39.36 | 15.92 | 74.43 | 30.2 | 15.78 | 57.38 | 40.33 | 39.75 | lower lateral or anterior |
| *Carcharias cuspidata* | SMNS 87457.303 | 12.57 | 24.15 | 24.78 | 1 | 5.04 | 1.76 | 1.55 | 25.18 | 23.05 | 9.76 | 100.2 | 19.3 | 9.34 | 33.49 | 25.42 | 25.22 | anterior |
| *Carcharias cuspidata* | SMNS 87457.304 | 18.89 | 39.59 | 40.29 | 1.1 | 9.89 | 3.33 | 1.81 | 41.48 | 37.65 | 14.47 | 84.61 | 27.93 | 14.12 | 53.71 | 39.55 | 38.16 | anterior |
| *Carcharias cuspidata* | SMNS 87457.305 | 15.21 | 30.11 | 30.83 | 1.2 | 7.2 | 2.32 | 1.66 | 31.47 | 28.72 | 12.61 | 69.45 | 21.08 | 10.23 | 40.34 | 32.07 | 32.06 | anterior |
| *Carcharias cuspidata* | SMNK Pal.6598a | 16.02 | 30.48 | 32.07 | 1 | 6.14 | 3.14 | - | - | 25.9 | 8.28 | 93.68 | 20.53 | 5.61 | 36.09 | 28 | 28.03 | anterior |
| *Carcharias cuspidata* | SMNK Pal.6598b | 15.95 | 12.97 | 13.15 | 21.24 | 4.5 | - | - | 18.46 | 12.06 | 8.62 | 140 | 22.36 | 6.54 | 19.51 | 16.66 | 12.84 | upper lateral |
| *Carcharias cuspidata* | SMNK Pal.6598c | 17.04 | 34 | 35 | 0.5 | 7.03 | 2.6 | 1.91 | 35.5 | 33 | 12.72 | 99.13 | 24.36 | 6.91 | 40.91 | 32 | 32.5 | anterior |
| *Carcharias cuspidata* | SMNK Pal.6598d | 15.23 | 13.03 | 13.84 | 11 | 4.35 | 2 | 2.11 | 16.43 | 11.39 | 8.68 | 134.31 | 20.99 | 4.1 | 17.13 | 14.05 | 11.36 | lower or upperlateral |
| *Carcharias cuspidata* | SMNK Pal.6598e | 16.94 | 17.77 | 18.93 | 8.57 | 4.35 | 1.8 | 2 | 20.83 | 16.4 | 9.3 | 131.64 | 24.59 | 5.39 | 23.16 | 18.21 | 16.05 | lower or upperlateral |
| *Carcharias cuspidata* | SMNK Pal.6598f | 19.08 | 37.57 | 38.81 | 1 | 7.04 | 4.22 | 2 | 39.29 | 35.41 | 14.2 | 113.5 | 29.4 | 9.79 | 47.36 | 37 | 35.71 | anterior |
| *Carcharias cuspidata* | SMNK Pal.6598g | 15.43 | 11.83 | 12.61 | 12.11 | 4.13 | 1.66 | 1.72 | 15.74 | 10.67 | 10.09 | 135.58 | 20.28 | 4.86 | 16.69 | 10.97 | 13.5 | upper lateral |
| *Carcharias gustrowensis* | SMNS 87457.311 | 8.47 | 10.24 | 11.01 | 3.54 | 2.21 | 2.6 | 1.38 | 11.2 | 9.41 | 5.86 | 118.61 | 12.96 | 4.01 | 14.25 | 10.2 | 9.62 | upper lateral |
| *Carcharias taurus* | NHMW 98950 | 5.64 | 11.13 | 11.38 | 7 | 2.39 | 1.31 | 0.7 | 12.32 | 10.56 | 4.49 | 88.33 | 8.75 | 5.27 | 16.4 | 11.4 | 10.7 | LA2L |
| *Carcharias taurus* | NHMW 98950 | 5.56 | 9.8 | 10.16 | 5.78 | 1.86 | 1.38 | 0.78 | 10.58 | 9.16 | 4.1 | 88.65 | 8.43 | 4.4 | 14.2 | 9.46 | 9.1 | LL1L |
| *Carcharias taurus* | NHMW 98950 | 5.12 | 6.61 | 7.06 | 1.45 | 1.47 | 1.12 | 0.76 | 7.14 | 6.16 | 3.57 | 102.03 | 7.88 | 3.62 | 10.23 | 6.4 | 6.4 | LL2L |
| *Carcharias taurus* | NHMW 98950 | 4.97 | 5.85 | 6.3 | 1 | 1.4 | 0.83 | 0.72 | 6.46 | 5.18 | 3.4 | 101.27 | 7.47 | 3.05 | 8.9 | 5.57 | 5.53 | LL3L |
| *Carcharias taurus* | NHMW 98950 | 4.44 | 4.69 | 5.05 | 0.8 | 1.26 | 0.7 | 0.82 | 5.35 | 4.27 | 2.97 | 109.37 | 6.25 | 2.43 | 7.12 | 4.65 | 4.27 | LL4L |
| *Carcharias taurus* | NHMW 98950 | 3.58 | 3.88 | 4.1 | 0.8 | 1.19 | 0.55 | 0.79 | 4.39 | 3.68 | 2.31 | 116.02 | 5.34 | 2.54 | 6.42 | 3.86 | 3.84 | LL5L |
| *Carcharias taurus* | NHMW 98950 | 3.55 | 2.49 | 3.02 | 1.2 | 0.96 | 0.75 | 0.8 | 3.08 | 2.31 | 1.93 | 114.54 | 4.64 | 2.48 | 4.97 | 2.53 | 2.31 | LL6L |
| *Carcharias taurus* | NHMW 98950 | 5.19 | 13.24 | 13.47 | 0.6 | 2.21 | 1.68 | 0.92 | 13.49 | 12.51 | 4.02 | 70.68 | 8.58 | 5.69 | 18.93 | 12.76 | 12.66 | LA1R |
| *Carcharias taurus* | NHMW 98950 | 6.29 | 13.16 | 13.2 | 5.88 | 2.7 | 1.76 | 0.93 | 13.95 | 12.3 | 4.82 | 80.99 | 9.94 | 5.39 | 18.55 | 13.06 | 12.41 | LA2R |
| *Carcharias taurus* | NHMW 98950 | 5.58 | 9.28 | 9.67 | 0.7 | 2.07 | 1.31 | 0.66 | 9.82 | 8.55 | 4.35 | 92.96 | 8.74 | 4.36 | 13.64 | 8.98 | 8.78 | LL1R |
| *Carcharias taurus* | NHMW 98950 | 5.15 | 7.14 | 7.63 | 0.7 | 1.58 | 1.14 | 0.86 | 7.64 | 6.76 | 3.77 | 100.77 | 8.35 | 3.72 | 10.86 | 7 | 6.96 | LL2R |
| *Carcharias taurus* | NHMW 98950 | 5.11 | 6.53 | 6.86 | 2.9 | 1.63 | 1.05 | 0.91 | 7.29 | 6 | 3.67 | 111.43 | 7.74 | 2.91 | 9.44 | 6.42 | 6.12 | LL3R |
| *Carcharias taurus* | NHMW 98950 | 4.7 | 5.64 | 5.87 | 3.32 | 1.44 | 0.97 | 0.71 | 6.36 | 5.12 | 3.14 | 105.53 | 6.68 | 2.99 | 8.63 | 5.6 | 5.19 | LL4R |
| *Carcharias taurus* | NHMW 98950 | 4.4 | 4.26 | 4.61 | 1.5 | 1.33 | 0.87 | 0.78 | 5.01 | 3.77 | 2.71 | 114.07 | 6.15 | 2.63 | 6.89 | 4.16 | 3.95 | LL5R |
| *Carcharias taurus* | NHMW 98950 | 3.58 | 3.06 | 3.34 | 7.3 | 1.14 | 0.71 | 0.52 | 3.79 | 2.38 | 2.39 | 122.15 | 4.72 | 1.87 | 4.93 | 2.96 | 2.62 | LL6R |
| *Carcharias taurus* | NHMW 98950 | 4.66 | 8.64 | 9.13 | 5.43 | 1.98 | 1.09 | 0.66 | 8.63 | 8.04 | 3.61 | 65.28 | 6.33 | 5.01 | 13.65 | 8.07 | 8.36 | UA1L |
| *Carcharias taurus* | NHMW 98950 | 6.16 | 8.9 | 8.99 | 6.2 | 2.25 | 1.4 | 0.84 | 10.13 | 8.11 | 4.44 | 106.02 | 9.68 | 3.93 | 12.83 | 9.09 | 8.32 | UA2L |
| *Carcharias taurus* | NHMW 98950 | 6.09 | 8.87 | 8.88 | 7.5 | 2.33 | 1.4 | 0.76 | 10.05 | 8.11 | 4.45 | 105.75 | 9.68 | 3.87 | 12.74 | 8.83 | 8.07 | UA3L |
| *Carcharias taurus* | NHMW 98950 | 5.86 | 6.23 | 6.49 | 11.29 | 1.75 | 1.34 | 0.81 | 7.62 | 5.39 | 3.7 | 114.65 | 8.73 | 3.1 | 9.33 | 6.45 | 5.57 | UL1L |
| *Carcharias taurus* | NHMW 98950 | 6.98 | 7.33 | 7.5 | 10.05 | 2.09 | 1.25 | 1.25 | 9.12 | 6.6 | 4.73 | 118.51 | 9.77 | 2.88 | 10.21 | 7.91 | 6.62 | UL2L |
| *Carcharias taurus* | NHMW 98950 | 6.11 | 6.13 | 6.32 | 12.25 | 1.75 | 1.06 | 1 | 7.74 | 5.62 | 4.23 | 114.9 | 8.52 | 3.41 | 9.54 | 6.82 | 5.7 | UL3L |
| *Carcharias taurus* | NHMW 98950 | 5.16 | 4.99 | 5.05 | 17.6 | 1.55 | 0.74 | 0.76 | 6.54 | 4.56 | 3.65 | 117.33 | 7.14 | 2.77 | 7.76 | 5.72 | 4.55 | UL4L |
| *Carcharias taurus* | NHMW 98950 | 4.5 | 3.87 | 3.95 | 21.53 | 1.57 | 0.71 | 0.75 | 5.31 | 3.29 | 3.09 | 119.94 | 6.49 | 2.15 | 6.02 | 4.41 | 3.26 | UL5L |
| *Carcharias taurus* | NHMW 98950 | 4.33 | 9.48 | 9.77 | 0.5 | 1.83 | 1.39 | 0.89 | 9.69 | 8.79 | 3.32 | 73.03 | 6.16 | 3.98 | 13.46 | 8.93 | 8.92 | UA1R |
| *Carcharias taurus* | NHMW 98950 | 7.02 | 10.65 | 10.9 | 7.89 | 2.19 | 1.77 | 1.27 | 12.14 | 9.9 | 4.9 | 105.75 | 10.85 | 4.62 | 15.27 | 10.79 | 10.03 | UA3R |
| *Carcharias taurus* | NHMW 98950 | 5.89 | 6.87 | 7.04 | 16.99 | 1.67 | 1.3 | 1.25 | 8.05 | 6.18 | 3.7 | 117.62 | 8.97 | 3 | 9.87 | 7 | 6.05 | UL1R |
| *Carcharias taurus* | NHMW 98950 | 7.34 | 8.37 | 8.71 | 10.45 | 2.2 | 1.35 | 1.27 | 9.33 | 7.39 | 4.9 | 110.65 | 10.45 | 3.47 | 11.84 | 8.34 | 7.41 | UL2R |
| *Carcharias taurus* | NHMW 98950 | 6.97 | 7.8 | 7.87 | 12.9 | 2.1 | 1.53 | 1.23 | 9.48 | 6.96 | 4.72 | 106.85 | 9.61 | 3.29 | 11.09 | 8.03 | 6.88 | UL3R |
| *Carcharias taurus* | NHMW 98950 | 5.37 | 5.37 | 5.51 | 14.35 | 1.59 | 0.95 | 0.86 | 6.6 | 4.78 | 3.75 | 115.34 | 7.44 | 2.52 | 7.89 | 5.64 | 4.7 | UL4R |
| *Carcharias taurus* | NHMW 98950 | 4.6 | 3.54 | 3.61 | 19.7 | 1.73 | - | - | 2.85 | 5 | 3.01 | 116.94 | 6.48 | 2.59 | 6.13 | 4.05 | 3 | UL5R |
| *Carcharomodus escheri* | MNU 071-20_10A | - | 31.7 | 32.9 | 19 | 13.76 | - | - | 40.9 | 31.43 | 24.71 | - | - | 10.3 | 42 | 39.58 | 31.58 | upper anterior |
| *Carcharomodus escheri* | MNU 071-20_10B | 21.91 | 28.2 | 27.8 | 14 | 11.82 | - | - | 33 | 25.54 | 19.76 | 130 | 24.7 | 6.8 | 35 | 30.31 | 25.63 | upper lateral |
| *Carcharomodus escheri* | MNU 071-20_10C | 22.4 | 28.1 | 27.4 | 18 | 11.14 | - | - | 32.9 | 24.72 | 19.14 | 135 | 24.42 | 6.8 | 34.9 | 26.67 | 25.57 | upper lateral |
| *Carcharomodus escheri* | MNU 071-20_10D | 23.37 | 24.4 | 25.7 | 14 | 10.46 | 1.7 | - | 29.8 | 21.46 | 18.11 | 125 | 27 | 6.6 | 31 | 27.65 | 23 | upper lateral |
| *Carcharomodus escheri* | MNU 071-20_10E | 22 | 23.3 | 24.9 | 17.1 | 10.6 | 2 | 1.84 | 29.5 | 21.3 | 18.31 | 150 | 26.9 | 6.2 | 29.5 | 25.16 | 20.6 | upper lateral |
| *Carcharomodus escheri* | MNU 071-20_10F | 19.3 | 21.4 | 21.5 | 13 | 9.51 | 2.1 | 1.82 | 23.9 | 18.87 | - | 135 | - | 6.2 | 27.6 | 24.83 | 20.71 | upper lateral |
| *Carcharomodus escheri* | MNU 071-20_10G | 16.36 | 14.5 | 14.4 | 20 | 6.54 | 1.2 | 0.81 | 20.4 | 12.51 | 12 | 136 | 18.1 | 4.9 | 19.4 | 16.32 | 13.52 | upper lateral |
| *Carcharomodus escheri* | MNU 071-20_10H | 18.62 | 26.9 | 27.3 | 11 | 11.86 | 2 | 2.5 | 30.2 | 17.58 | 14.38 | 142.8 | 21.96 | 5.76 | 32.66 | 17.41 | 21.5 | upper lateral |
| *Carcharomodus escheri* | MNU 071-20_10I | 20.8 | 28.9 | 29.5 | 17 | 12.11 | 2.52 | 2.75 | 32 | 26.94 | 18.72 | 135 | 25 | 7.9 | 36.8 | 30.18 | 25.21 | upper lateral |
| *Carcharomodus escheri* | MNU 071-20_11A | 17.97 | 27.3 | 28.1 | 7.2 | 8.32 | 1.8 | 1.64 | 30 | 24.19 | 14.07 | 110 | 22 | 7.6 | 34.9 | 24.88 | 23.44 | lower anterior |
| *Carcharomodus escheri* | MNU 071-20_11C | 22.65 | 21.23 | 24.5 | 3 | 8.39 | 2.7 | - | 24.7 | 21.23 | 22.65 | 145 | 25.5 | 8.97 | 30.2 | 21.5 | 51.4 | lower lateral |
| *Carcharomodus escheri* | MNU 071-20_11D | 20.15 | 17 | 19.5 | 1 | 5.98 | 3.1 | 2.64 | 21 | 14.72 | 11.4 | 125 | 20.1 | 6 | 23 | 15.62 | 15.3 | lower lateral |
| *Carcharomodus escheri* | MNU 071-20_11E | 19.43 | 17.82 | 20.3 | 1 | 6.77 | 3 | 3.58 | 20.8 | 14.81 | 14.96 | 130 | 22.2 | 5.68 | 23.5 | 15.43 | 15.23 | lower lateral |
| *Carcharomodus escheri* | MNU 071-20_11F | 17.61 | 16.45 | 19.3 | 2 | 5.82 | 3 | 3.11 | 19.7 | 14.01 | 12 | 125 | 19.5 | 5.55 | 22 | 15.4 | 14.72 | lower lateral |
| *Carcharomodus escheri* | MNU 071-20_11G | 15.2 | 13.44 | 15 | 3 | 5 | 2.8 | 2.24 | 16.2 | 11.88 | 10.5 | 137 | 16.1 | 4.96 | 18.4 | 12.54 | 12.45 | lower lateral |
| *Carcharomodus escheri* | MNU 071-20_11H | 12.34 | 10.83 | 11.7 | 3 | 3.66 | 2.2 | 2.2 | 13.4 | 9.51 | 7.3 | 140 | 12.6 | 3.97 | 14.8 | 9.79 | 9.3 | lower lateral |
| INDETERMINATE | NRM-PZ P15929 | 7.95 | 8.23 | 8.9 | 3.96 | 2.29 | 1.83 | 1.27 | 9.46 | 7.44 | 5.3 | - | - | 3.68 | 11.91 | 8.14 | 7.81 | - |
| INDETERMINATE | NRM-PZ P15837 | - | 11.37 | - | 8.85 | 3.5 | 3.2 | 2.07 | 13.8 | 9.26 | 6.78 | - | - | 5.06 | 16.43 | 10.67 | 9.38 | - |
| INDETERMINATE | NRM-PZ P15775 | - | 8.62 | - | 4.19 | 2.62 | 1.89 | 1.32 | 9.86 | 7.57 | 5.11 | - | - | 3.51 | 12.13 | 8.48 | 7.49 | - |
| *Lamna nasus* | ZC UniVie_2016_1 | 6.65 | 4.9 | 5.37 | 10.76 | 2.32 | 0.95 | 1.1 | 6.5 | 4.11 | 4.31 | 122.16 | 8.52 | 3.88 | 8.78 | 5.15 | 4.19 | UL7L |
| *Lamna nasus* | ZC UniVie_2016_1 | 6.44 | 4.6 | 5.12 | 5.77 | 1.89 | 0.7 | 1.12 | 6.12 | 3.98 | 4.13 | 106.16 | 8.33 | 4.48 | 9.08 | 4.84 | 4.25 | UL7R |
| *Lamna nasus* | ZC UniVie_2016_1 | 5.69 | 5.66 | 5.98 | 6.66 | 2.08 | 0.87 | 0.86 | 6.7 | 4.92 | 3.8 | 109.1 | 7.14 | 3.49 | 9.15 | 5.56 | 5 | LL7L |
| *Lamna nasus* | ZC UniVie_2016_1 | 6.39 | 5.6 | 6.21 | 0.4 | 2.02 | 1.01 | 0.88 | 6.55 | 5.09 | 4.44 | 112.95 | 6.98 | 3.74 | 9.34 | 5.63 | 5.39 | LL7R |
| *Lamna nasus* | ZC UniVie_2016_1 | 8.23 | 6.23 | 6.74 | 11.07 | 2.51 | 1.44 | 1.51 | 8.37 | 5.42 | 5.05 | 111.84 | 8.62 | 3.56 | 9.79 | 6.68 | 5.59 | UL6L |
| *Lamna nasus* | ZC UniVie_2016_1 | 7.49 | 5.37 | 5.93 | 8.22 | 2.59 | 0.75 | 1.24 | 7.25 | 5.06 | 4.94 | 125.98 | 9.87 | 4.43 | 9.8 | 6.2 | 5.29 | UL6R |
| *Lamna nasus* | ZC UniVie_2016_1 | 7.79 | 6.58 | 7.54 | 0.45 | 2.62 | 0.98 | 1.2 | 7.72 | 6.18 | 5.25 | 122 | 9.06 | 4.15 | 10.73 | 6.78 | 6.7 | LL6R |
| *Lamna nasus* | ZC UniVie_2016_1 | 7.81 | 7.15 | 7.88 | 3.61 | 2.24 | 1.41 | 1.54 | 8.41 | 6.66 | 4.56 | 116.09 | 8.87 | 4.38 | 11.53 | 7.18 | 6.87 | LL6L |
| *Lamna nasus* | ZC UniVie_2016_1 | 9.18 | 7.31 | 7.79 | 19.11 | 3.02 | 1.26 | 1.55 | 9.76 | 6.94 | 5.87 | 120.71 | 12.41 | 4.46 | 11.77 | 8.41 | 7.09 | UL1L |
| *Lamna nasus* | ZC UniVie_2016_1 | 8.76 | 7.61 | 8.28 | 5.4 | 2.68 | 1.37 | 1.35 | 9.3 | 6.78 | 5.84 | 110.93 | 9.96 | 4.37 | 11.98 | 7.74 | 7.13 | UL5L |
| *Lamna nasus* | ZC UniVie_2016_1 | 9.1 | 7.99 | 8.95 | 1.2 | 2.71 | 1.24 | 1.67 | 9.26 | 7.49 | 5.77 | 106.91 | 10.54 | 4.33 | 12.32 | 8.15 | 7.88 | LL5L |
| *Lamna nasus* | ZC UniVie_2016_1 | 8.09 | 7.42 | 8.07 | 4.49 | 2.78 | 1.23 | 1.42 | 9.15 | 6.74 | 5.6 | 108.58 | 10.33 | 4.94 | 12.36 | 7.93 | 6.96 | UL5R |
| *Lamna nasus* | ZC UniVie_2016_1 | 9.47 | 8.27 | 9.08 | 0.55 | 3 | 1.59 | 1.59 | 9.88 | 7.49 | 5.77 | 122.78 | 10.65 | 4.5 | 12.77 | 8.3 | 7.8 | LL5R |
| *Lamna nasus* | ZC UniVie_2016_1 | 8.89 | 8.29 | 8.96 | 8.28 | 3 | 1.19 | 1.13 | 10.02 | 7.72 | 6.5 | 126.48 | 11.82 | 4.57 | 12.86 | 8.89 | 8.1 | UL4L |
| *Lamna nasus* | ZC UniVie_2016_1 | 9.93 | 7.76 | 8.79 | 9.47 | 3.17 | 1.62 | 1.83 | 9.89 | 7.11 | 6 | 110.4 | 13 | 5.15 | 12.91 | 8.07 | 7.3 | UL1R |
| *Lamna nasus* | ZC UniVie_2016_1 | 9.29 | 8.2 | 9.22 | 3.91 | 3.11 | 1.28 | 1.52 | 9.89 | 7.82 | 6.43 | 120.78 | 12.45 | 4.78 | 12.98 | 8.82 | 8.25 | UL4R |
| *Lamna nasus* | ZC UniVie_2016_1 | 9.43 | 8.48 | 9.52 | 0.61 | 2.7 | 1.37 | 1.7 | 10 | 7.72 | 5.9 | 121.15 | 11 | 4.52 | 13 | 8.37 | 8.33 | LL4R |
| *Lamna nasus* | ZC UniVie_2016_1 | 9.73 | 8.38 | 8.94 | 14.22 | 2.85 | 1.65 | 1.55 | 10.49 | 7.73 | 6.59 | 124.95 | 13.95 | 4.79 | 13.17 | 9.16 | 7.93 | UL2L |
| *Lamna nasus* | ZC UniVie_2016_1 | 9.1 | 8.7 | 9.77 | 0.8 | 2.9 | 1.5 | 1.56 | 9.84 | 8.27 | 5.93 | 126.44 | 10.98 | 4.67 | 13.37 | 8.65 | 8.7 | LL4L |
| *Lamna nasus* | ZC UniVie_2016_1 | 9.2 | 8.89 | 9.79 | 4.22 | 2.87 | 1.12 | 1.4 | 10.38 | 8.38 | 6.41 | 123.41 | 12.56 | 4.81 | 13.7 | 9.31 | 8.81 | UL3R |
| *Lamna nasus* | ZC UniVie_2016_1 | 9.63 | 9.04 | 9.97 | 0.4 | 3.12 | 1.38 | 1.16 | 10.3 | 8.3 | 6.55 | 118.49 | 12.03 | 4.73 | 13.77 | 9.1 | 8.86 | LL3R |
| *Lamna nasus* | ZC UniVie_2016_1 | 9.69 | 9.53 | 10.71 | 0.75 | 3 | 1.19 | 1.44 | 10.81 | 9.04 | 6.65 | - | 11.69 | 4.5 | 14.03 | 9.94 | 9.67 | LL3L |
| *Lamna nasus* | ZC UniVie_2016_1 | 9.6 | 9.45 | 10.36 | 6.34 | 2.83 | 1.3 | 2.02 | 10.72 | 9.04 | 6.5 | 123.86 | 12.63 | 5.05 | 14.5 | 9.86 | 9.43 | UL3L |
| *Lamna nasus* | ZC UniVie_2016_1 | 10.3 | 9.45 | 10.68 | 0.8 | 3.31 | 1.65 | 1.32 | 10.78 | 8.81 | 7.03 | 114.46 | 13.32 | 5.34 | 14.79 | 9.52 | 9.44 | LL2L |
| *Lamna nasus* | ZC UniVie_2016_1 | 10 | 9.19 | 10.55 | 0.5 | 3.21 | 1.45 | 1.4 | 10.6 | 8.7 | 6.76 | 124.4 | 12.47 | 5.89 | 15.08 | 9.58 | 9.5 | LL2R |
| *Lamna nasus* | ZC UniVie_2016_1 | 9.94 | 10.03 | 10.44 | 7.16 | 2.87 | 1.7 | 1.65 | 11.69 | 9.57 | 6.89 | 133.24 | 13.39 | 5.69 | 15.72 | 10.54 | 9.8 | UL2R |
| *Lamna nasus* | ZC UniVie_2016_1 | 9.85 | 10.05 | 10.75 | 9.55 | 3.15 | 1.29 | 1.31 | 12.28 | 9.71 | 6.84 | 128.66 | 14.17 | 5.76 | 15.81 | 11.08 | 9.87 | UA2R |
| *Lamna nasus* | ZC UniVie_2016_1 | 10.13 | 9.97 | 10.85 | 3.28 | 2.97 | 1.5 | 1.38 | 11.6 | 9.8 | 6.75 | 116.9 | 13.75 | 5.94 | 15.91 | 10.8 | 10.32 | LL1L |
| *Lamna nasus* | ZC UniVie_2016_1 | 8.67 | 10.02 | 11.31 | 6.99 | 2.97 | 1.7 | 1.41 | 10.56 | 9.51 | 5.61 | 114.2 | 12.2 | 6.03 | 16.05 | 9.68 | 10.23 | LA1L |
| *Lamna nasus* | ZC UniVie_2016_1 | 9.41 | 10.2 | 10.63 | 5.93 | 2.9 | 1.55 | 1.22 | 11.62 | 9.53 | 6.34 | 112.3 | 13.47 | 6.08 | 16.28 | 10.7 | 9.87 | LA2L |
| *Lamna nasus* | ZC UniVie_2016_1 | 10.31 | 10.72 | 11.06 | 4.81 | 3.24 | 1.06 | 1.14 | 12.86 | 10.71 | 7.45 | 114.21 | 14.28 | 5.75 | 16.47 | 12.14 | 10.84 | UA2L |
| *Lamna nasus* | ZC UniVie_2016_1 | 9.28 | 10.86 | 11.38 | 2.7 | 3.07 | 1.38 | 1.26 | 11.61 | 10.25 | 5.78 | 99.81 | 10.66 | 5.97 | 16.83 | 10.61 | 10.54 | LL1R |
| *Lamna nasus* | ZC UniVie_2016_1 | 8.58 | 11.86 | 12.98 | 2.81 | 2.96 | 2.06 | 1.35 | 12.3 | 10.92 | 6.12 | 94.21 | 11.67 | 5.44 | 17.3 | 11.17 | 11.6 | LA1R |
| *Lamna nasus* | ZC UniVie_2016_1 | 10 | 11.16 | 12.56 | 2.23 | 3.87 | 0.96 | 1.3 | 12.13 | 11.14 | 7.57 | 97.97 | 12.93 | 6.24 | 17.4 | 11.67 | 11.98 | UA1L |
| *Lamna nasus* | ZC UniVie_2016_1 | 8.77 | 11.7 | - | 5.63 | 3.22 | 1.38 | 1.06 | 11.28 | 10.52 | 6.45 | - | - | 5.74 | 17.44 | 11.74 | 10.76 | UA1R |
| *Lamna nasus* | ZC UniVie_2016_1 | 10.28 | 11.71 | 12.76 | 0.8 | 3.52 | 1.4 | 1.47 | 13.05 | 11.08 | 7.21 | 117.5 | 14.72 | 6.39 | 18.1 | 11.86 | 11.79 | LA2R |
| *Lamna nasus* | NHMW 93611 | 6.8 | 9.1 | 9.2 | 10.72 | 2.48 | 0.75 | 0.77 | 10.41 | 8.68 | 5.19 | 96.19 | 9.1 | 2.86 | 11.96 | 9.58 | 8.79 | UA1L |
| *Lamna nasus* | NHMW 93611 | 6.88 | 7.98 | 8.03 | 14.18 | 2.42 | 0.94 | 0.82 | 9.88 | 7.62 | 5.46 | 102.91 | 8.19 | 2.94 | 10.92 | 9.14 | 7.63 | UA2L |
| *Lamna nasus* | NHMW 93611 | 5.94 | 5.6 | 5.5 | 24 | 2.12 | 0.85 | 1.02 | 8.02 | 5.16 | 4.09 | 115.12 | 7.91 | 2.55 | 8.15 | 6.9 | 5.18 | UL1L |
| *Lamna nasus* | NHMW 93611 | 5.72 | 5.29 | 5.35 | 20.15 | 2.11 | 0.75 | 0.82 | 7.16 | 4.86 | 4.17 | 108.21 | 7.62 | 2.46 | 7.75 | 6.32 | 4.94 | UL2L |
| *Lamna nasus* | NHMW 93611 | 6.3 | 6.42 | 6.61 | 15.22 | 2.15 | 1.01 | 0.95 | 8.29 | 5.95 | 4.5 | 119.51 | 7.67 | 2.53 | 8.95 | 7.32 | 5.88 | UL3L |
| *Lamna nasus* | NHMW 93611 | 5.78 | 6.06 | 6.25 | 12.08 | 2 | 0.86 | 0.78 | 7.28 | 5.52 | 4.26 | 118.64 | 7.34 | 2.3 | 8.36 | 6.47 | 5.65 | UL4L |
| *Lamna nasus* | NHMW 93611 | 5.12 | 5.04 | 5.32 | 9.22 | 1.81 | 0.97 | 0.81 | 5.89 | 4.57 | 3.65 | 106.63 | 6.15 | 2.29 | 7.33 | 5.15 | 4.62 | UL5L |
| *Lamna nasus* | NHMW 93611 | 4.49 | 4.38 | 4.57 | 9.9 | 1.55 | 0.68 | 0.53 | 5.28 | 3.88 | 3.38 | 110.41 | 5.27 | 2.22 | 6.6 | 4.67 | 4.02 | UL6L |
| *Lamna nasus* | NHMW 93611 | 3.86 | 3.57 | 3.9 | 5.43 | 1.25 | 0.55 | 0.68 | 4.28 | 3.1 | 2.64 | 95.11 | 4.7 | 2.06 | 5.63 | 3.49 | 3.23 | UL7L |
| *Lamna nasus* | NHMW 93611 | 6.86 | 8.46 | 8.67 | 12.65 | 2.44 | 0.89 | 0.87 | 9.97 | 8.1 | 5.11 | 112.28 | 8.35 | 2.98 | 11.44 | 9.15 | 8.15 | UA1R |
| *Lamna nasus* | NHMW 93611 | 6.85 | 8.22 | 8.11 | 19.14 | 2.52 | 0.71 | 0.77 | 10.43 | 7.95 | 5.4 | 105.39 | 8.31 | 2.95 | 11.17 | 9.45 | 7.77 | UA2R |
| *Lamna nasus* | NHMW 93611 | 5.54 | 5.24 | 5.23 | 20.7 | 2.02 | 0.73 | 0.71 | 7.14 | 4.93 | 3.87 | 119.21 | 8.58 | 3.29 | 8.53 | 6.4 | 4.83 | UL1R |
| *Lamna nasus* | NHMW 93611 | 6.56 | 6.7 | 6.71 | 20.77 | 2.51 | 0.8 | 0.81 | 9 | 6.36 | 4.74 | 111.64 | 9.25 | 3.18 | 9.88 | 7.98 | 6.25 | UL2R |
| *Lamna nasus* | NHMW 93611 | 6.05 | 6.3 | 6.39 | 16.11 | 2.06 | 0.88 | 0.95 | 7.96 | 5.85 | 4.49 | 108.79 | 8.01 | 2.94 | 9.24 | 7.17 | 5.92 | UL3R |
| *Lamna nasus* | NHMW 93611 | 5.41 | 5.79 | 6.11 | 8.62 | 1.95 | 0.65 | 0.71 | 6.75 | 5.34 | 4.04 | 104.86 | 6.82 | 2.63 | 8.42 | 6.03 | 5.5 | UL4R |
| *Lamna nasus* | NHMW 93611 | 5.26 | 5.26 | 5.52 | 10.61 | 1.8 | 0.78 | 0.65 | 6.27 | 4.78 | 3.85 | 103.82 | 5.97 | 2.39 | 7.65 | 5.54 | 4.88 | UL5R |
| *Lamna nasus* | NHMW 93611 | 3.93 | 3.45 | 3.47 | 9.94 | 1.41 | 0.58 | 0.68 | 4.27 | 3.2 | - | 108.22 | 4.63 | 1.87 | 5.32 | - | 3.24 | UL7R |
| *Lamna nasus* | NHMW 93611 | 6.74 | 10.22 | 10.72 | 0.78 | 2.09 | 1.21 | 0.9 | 10.78 | 9.72 | 5.09 | 115.25 | 8.47 | 2.71 | 12.93 | 10.05 | 10.22 | LA1L |
| *Lamna nasus* | NHMW 93611 | 7.5 | 9.12 | 9.46 | 5.33 | 2.34 | 1.13 | 0.87 | 10.35 | 8.46 | 5.82 | 112.6 | 8.84 | 2.72 | 11.84 | 9.36 | 8.78 | LA2L |
| *Lamna nasus* | NHMW 93611 | 6.48 | 7.53 | 7.96 | 4.29 | 1.99 | 0.87 | 0.77 | 8.37 | 7.13 | 4.71 | 121.4 | 7.44 | 2.73 | 10.26 | 7.68 | 7.39 | LL1L |
| *Lamna nasus* | NHMW 93611 | 6.29 | 6.44 | 6.98 | 4.36 | 1.71 | 0.99 | 0.77 | 7.34 | 6.02 | 4.21 | 112.12 | 7.79 | 2.99 | 9.43 | 6.66 | 6.24 | LL2L |
| *Lamna nasus* | NHMW 93611 | 6.01 | 6.35 | 7.05 | 0.4 | 1.71 | 0.86 | 0.88 | 7.02 | 6.02 | 4.33 | 102.36 | 7.5 | 2.88 | 9.23 | 6.36 | 6.4 | LL3L |
| *Lamna nasus* | NHMW 93611 | 5.46 | 5.54 | 6 | 2.28 | 1.59 | 0.75 | 0.89 | 6.23 | 5.18 | 3.85 | 118 | 7.42 | 2.65 | 8.19 | 5.6 | 5.47 | LL4L |
| *Lamna nasus* | NHMW 93611 | 5.61 | 5.7 | 6.27 | 1.99 | 1.51 | 0.9 | 1 | 6.49 | 5.44 | 3.38 | 109.28 | 6.21 | 2.49 | 8.19 | 5.76 | 5.59 | LL5L |
| *Lamna nasus* | NHMW 93611 | 4.54 | 4.41 | 4.92 | 0.3 | 1.24 | 0.55 | 0.77 | 5.01 | 4.19 | 3.1 | 104.24 | 5.26 | 2.26 | 6.67 | 4.48 | 4.49 | LL6L |
| *Lamna nasus* | NHMW 93611 | 3.98 | 3.7 | 4.05 | 1 | 1.09 | 0.46 | 0.58 | 4.14 | 3.53 | 2.58 | 88.85 | 4.27 | 2.28 | 5.98 | 3.67 | 3.62 | LL7L |
| *Lamna nasus* | NHMW 93611 | 6.69 | 10.52 | 11.42 | 4.29 | 2.12 | 1.45 | 0.8 | 11.67 | 11.01 | 5.13 | 101.93 | 8.63 | 4.3 | 14.82 | 11.12 | 10.82 | LA1R |
| *Lamna nasus* | NHMW 93611 | 6.36 | 8.5 | 8.68 | 5.75 | 2.13 | 0.95 | 0.76 | 9.74 | 8.06 | 4.83 | 98.9 | 7.77 | 3.73 | 12.23 | 8.92 | 8.21 | LA2R |
| *Lamna nasus* | NHMW 93611 | 5.77 | 6.89 | 7.35 | 1 | 1.76 | 0.98 | 0.88 | 7.68 | 6.46 | 4.13 | - | 6.81 | 2.95 | 9.84 | 6.92 | 6.56 | LL1R |
| *Lamna nasus* | NHMW 93611 | 5.99 | 6.5 | 6.86 | 1.1 | 1.68 | 1.02 | 0.84 | 7.45 | 6.14 | 4.13 | 110.41 | 7.89 | 3.06 | 9.56 | 6.59 | 6.25 | LL2R |
| *Lamna nasus* | NHMW 93611 | 6.33 | 7.03 | 7.52 | 1 | 1.71 | 1.02 | 0.97 | 7.75 | 6.55 | 4.45 | 115.03 | 7.83 | 2.83 | 9.86 | 6.9 | 6.8 | LL3R |
| *Lamna nasus* | NHMW 93611 | 6.4 | 6.58 | 7.27 | 1 | 1.72 | 1.16 | 0.95 | 7.4 | 5.98 | 4.33 | 112.84 | 7.67 | 2.71 | 9.29 | 6.38 | 6.35 | LL4R |
| *Lamna nasus* | NHMW 93611 | 6.7 | 6.74 | 7.35 | 1 | 1.89 | 1.07 | 1.15 | 7.88 | 6.27 | 4.17 | 115.02 | 8.05 | 3.17 | 9.91 | 6.71 | 6.45 | LL5R |
| *Lamna nasus* | NHMW 93611 | 4.54 | 4.59 | 4.92 | 1.8 | 1.18 | 0.81 | 0.76 | 5.43 | 4.22 | 2.96 | 103.39 | 5 | 2.31 | 6.9 | 4.73 | 4.36 | LL6R |
